# Renewable Energy Literacy and Multidimensional Energy-Saving Behavior in Indonesia: The Roles of Personal Energy Values and Energy Attitudes

**DOI:** 10.12688/f1000research.185821.1

**Published:** 2026-07-29

**Authors:** Rahmadi Budiman, Dewi Kusuma Wardani, Aniek Hindrayani, Muhammad Sabandi

**Affiliations:** 1 Department of Economics Education, Faculty of Teacher Training and Education, Sebelas Maret University, Surakarta, Central Java, Indonesia

**Keywords:** renewable energy literacy; energy-saving behavior; energy demand management; energy transition; behavioral determinants; PLS-SEM; Indonesia

## Abstract

**Background:**

Demand-side behavioral change is an important component of sustainable energy transitions, yet limited evidence explains how renewable energy literacy translates into distinct forms of energy-saving behavior in emerging economies.

**Methods:**

This cross-sectional quantitative explanatory study surveyed 195 members of the Indonesia Fuel Cell and Hydrogen Energy Association. Renewable energy literacy was modeled as the predictor, personal energy values and energy attitudes as mediators, and energy-saving behavior as a multidimensional second-order construct comprising curtailment, efficiency, and pro-social behaviors. Data were analyzed using Partial Least Squares Structural Equation Modeling in SmartPLS 4 with 5,000 bootstrap resamples.

**Results:**

Renewable energy literacy was positively associated with energy-saving behavior directly and indirectly through personal energy values and energy attitudes. The total effect of renewable energy literacy on energy-saving behavior was substantial (β = 0.599, p < .001), while the direct effect was smaller (β = 0.182, p = .023). Personal energy values (indirect β = 0.241, p < .001; VAF = 57%) and energy attitudes (indirect β = 0.176, p < .001; VAF = 49%) both partially mediated this relationship. Mediated effects were stronger for pro-social and curtailment behaviors than for efficiency behavior.

**Conclusions:**

Literacy-based interventions can support energy conservation by strengthening conservation-oriented values and favorable attitudes. However, efficiency-related actions may require complementary economic or policy instruments because they are more likely to involve capital investment and structural constraints.

## Introduction

The global transition toward renewable energy systems requires coordinated action on both the supply and demand sides of energy markets. While governments, international institutions, and the private sector have directed substantial resources toward expanding renewable energy infrastructure and reforming energy policy frameworks, the ultimate effectiveness of these supply-side measures depends critically on whether individual and organizational energy users adopt conservation behaviors that reduce aggregate demand ( Yildiz, 2026; IEA, 2023). Behavioral change at the household and organizational level is increasingly recognized as an important complement to supply-side investment in achieving energy transition objectives. The importance of demand-side management is further supported by evidence that supply-side renewable energy expansion alone is insufficient to meet emissions reduction commitments; sustained reductions in energy consumption intensity are required alongside the decarbonization of energy supply ( Yildiz, 2026; Berrich, Mafakheri, & Dabbou, 2024).

Household and organizational energy consumption constitute particularly significant domains for demand-side management. In the Asia-Pacific region, rapidly developing economies such as Vietnam and Indonesia are experiencing pronounced growth in residential and commercial energy consumption, driven by rising incomes, accelerating urbanization, and increased ownership of energy-consuming appliances and technologies. Duy, Cassells, and Hanly (2023) demonstrated that household electricity consumption in Vietnam grew by over 20% in just two years, driven by income growth and lifestyle change, and that behavioral and structural interventions (including household adoption of micro-renewable energy systems) can generate meaningful reductions in residential energy expenditure. This evidence underscores the economic significance of demand-side behavioral change as a complement to renewable energy supply expansion in emerging economies ( Duy et al., 2023). In Indonesia, these dynamics are compounded by the country’s ambitious net-zero commitments and the structural challenge of transitioning an economy that remains heavily dependent on fossil fuel-based energy ( IEA, 2023; Hardi et al., 2024).

Among the behavioral determinants of household and professional energy use that have attracted growing scholarly attention, renewable energy literacy (REL) occupies an increasingly prominent position. From an energy economics perspective, REL represents a form of energy-related human capital: the knowledge, understanding, and competencies that equip individuals with the informational basis for informed energy decision-making and conservation behavior ( DeWaters & Powers, 2013). This framing parallels the established relationship between financial literacy and household financial behavior: just as financially literate individuals are better positioned to make informed savings and investment decisions ( Mireku, Appiah, & Agana, 2023), energy-literate individuals are better equipped to identify and act upon energy conservation opportunities in their professional and household lives. Empirical evidence consistently documents that higher levels of energy literacy are positively associated with conservation-oriented behaviors across diverse national and institutional contexts ( Lee, Lee, Altschuld, & Pan, 2015; Pothitou, Varga, Kolios, & Gu, 2015; Santillan & Cedano, 2023).

However, a persistent knowledge–behavior gap presents a practical challenge for demand-side energy management policy: individuals possessing adequate energy knowledge do not always translate such understanding into corresponding conservation practices, particularly at the household level where energy costs, technology access, and behavioral norms interact to shape energy decisions ( Brounen, Kok, & Quigley, 2013; do Paço & Varejo, 2010). Pothitou et al. (2015) demonstrated that while environmental knowledge and predisposition were significantly correlated with energy attitudes, habits, and behaviors in their household study, the association between knowledge and actual conservation behavior was considerably attenuated relative to the attitude–behavior link. do Paço and Varejo (2010), in their analysis of energy-saving behavior among Portuguese consumers, found that even among environmentally concerned respondents, economic factors (particularly electricity cost) constituted the primary differentiator between energy savers and non-savers. These findings suggest that information provision alone is insufficient to close the behavioral gap in energy demand management, and that motivational and attitudinal mechanisms must be understood and targeted to generate effective conservation outcomes.

Two theoretical frameworks provide complementary accounts of these behavioral mechanisms. Value–Belief–Norm (VBN) theory ( Stern, 2000) proposes that pro-environmental behavior is generated through a motivational sequence in which underlying value orientations (particularly biospheric and altruistic orientations) shape ecological beliefs and personal norms, which in turn motivate behavioral commitment. In the energy domain, such value-based motivations have been shown to constitute important antecedents of conservation behavior across organizational and professional contexts ( Al Mamun et al., 2022; Kim & Kim, 2024). The Theory of Planned Behavior (TPB; Ajzen, 1991) independently identifies attitudes as important proximal determinants of behavioral intention and action. Evidence from multiple national and institutional contexts confirms that favorable attitudes toward energy conservation are associated with stronger engagement in conservation practices ( Puiu et al., 2025; Xuan et al., 2023). Together, VBN and TPB suggest that the behavioral gap between energy literacy and conservation action may be mediated by values-based motivational processes and attitude-driven evaluative mechanisms.

These issues are particularly salient in the Indonesian context. Indonesia is simultaneously one of Southeast Asia’s largest and most rapidly growing economies and one of the most critical emerging energy markets for global climate policy. The country has committed to achieving net-zero emissions by 2060 and is navigating the complex economic and institutional reforms required to shift from fossil fuel dependence toward sustainable energy systems ( IEA, 2023; IESR, 2025). In this context, understanding the behavioral factors that encourage energy conservation is increasingly important, as demand-side actions constitute a critical complement to technological innovation and energy policy interventions.

Despite growing evidence that renewable energy literacy promotes conservation-oriented behavior, an important theoretical and policy question remains unresolved: how does energy literacy translate into actual conservation action, particularly when conservation behaviors differ in their economic requirements and structural constraints? Existing research consistently reports positive associations between literacy and energy-saving behavior, yet the observed effects are often modest, suggesting that knowledge alone is insufficient to generate sustained behavioral change. This persistent knowledge–behavior gap indicates the need to examine the motivational and evaluative mechanisms through which literacy influences conservation decisions.

While Appiah et al. (2023) demonstrated that energy-related values and attitudes partially mediate the relationship between energy literacy and energy-saving behavior, two important questions remain unaddressed. First, their study treats energy-saving behavior as a composite outcome, which precludes examination of whether literacy-driven pathways operate with equal strength across behaviorally distinct conservation domains. Second, evidence from professional energy communities in emerging economies undergoing active energy transition (a context that differs substantially from student and general household samples) remains limited. The present study addresses both gaps by operationalizing energy-saving behavior as a multidimensional second-order construct comprising curtailment, efficiency, and pro-social behavioral subdimensions, and by drawing on a professional renewable energy community in Indonesia as its study population.

Furthermore, energy-saving behavior has frequently been treated as a homogeneous construct despite encompassing multiple behavioral domains with distinct economic and behavioral characteristics. Curtailment behaviors typically require routine behavioral adjustments, efficiency behaviors often involve financial investment and technological adoption, whereas pro-social behaviors depend on social engagement and advocacy. Consequently, the pathways linking renewable energy literacy to these behavioral domains may not operate uniformly. Understanding such differences is particularly important in emerging economies undergoing energy transition, where economic constraints and institutional conditions may shape the effectiveness of literacy-based conservation interventions.

To address these gaps, this study examines the direct and indirect effects of renewable energy literacy on multidimensional energy-saving behavior through personal energy values and energy attitudes. By integrating VBN theory and TPB within a unified framework and distinguishing among curtailment, efficiency, and pro-social energy behaviors, the study advances understanding of literacy-driven behavioral mechanisms and provides new evidence relevant to demand-side energy management and behavioral energy economics in Indonesia.

Building on these gaps, the present study pursues three interconnected research objectives: (a) to determine whether REL exerts a direct effect on multidimensional energy-saving behavior; (b) to test whether personal energy values and energy attitudes function as behavioral mediators linking REL and ESB; and (c) to assess whether the magnitude of these mediated effects differs across the subdimensions of energy-saving behavior, specifically curtailment, efficiency, and pro-social behavioral subdimensions. Four hypotheses are advanced:

H1: Renewable energy literacy is positively associated with energy-saving behavior.

H2: Personal energy values mediate the positive relationship between renewable energy literacy and energy-saving behavior.

H3: Energy attitudes mediate the positive relationship between renewable energy literacy and energy-saving behavior.

H4: The mediated effects of renewable energy literacy on energy-saving behavior are stronger for curtailment and pro-social behaviors than for efficiency behaviors.

This study contributes to the behavioral energy economics and energy conservation literature in three interconnected ways. First, it extends prior research by examining whether renewable energy literacy exerts differential effects across distinct behavioral domains of energy-saving behavior. By operationalizing ESB as a multidimensional second-order construct comprising curtailment, efficiency, and pro-social subdimensions, the study moves beyond the common treatment of ESB as a homogeneous outcome and provides evidence on whether literacy-driven pathways are equally effective across these domains. Second, it integrates VBN theory and TPB within a unified PLS-SEM framework, treating personal energy values as a motivational pathway and energy attitudes as an evaluative pathway, and tests both simultaneously. Third, it provides evidence from a professional renewable energy community in Indonesia, an emerging economy in active energy transition, offering insights into how literacy-driven behavioral mechanisms operate among actors with substantive sectoral knowledge and professional exposure to renewable energy systems.

## Methods

### Study design and conceptual framework

This study employs a cross-sectional, quantitative explanatory design to test the hypothesized structural relationships among renewable energy literacy, personal energy values, energy attitudes, and energy-saving behavior. The conceptual framework posits that REL exerts both direct and indirect effects on ESB, with personal energy values (grounded in VBN theory) and energy attitudes (grounded in TPB) functioning as behavioral mediators. Energy-saving behavior is specified as a multidimensional second-order construct comprising three first-order subdimensions, namely curtailment behavior (ESB_CUR), efficiency behavior (ESB_EFF), and pro-social energy action (ESB_PRO), enabling simultaneous assessment of overall energy conservation behavior and its constituent behavioral domains.

**Figure 1.**
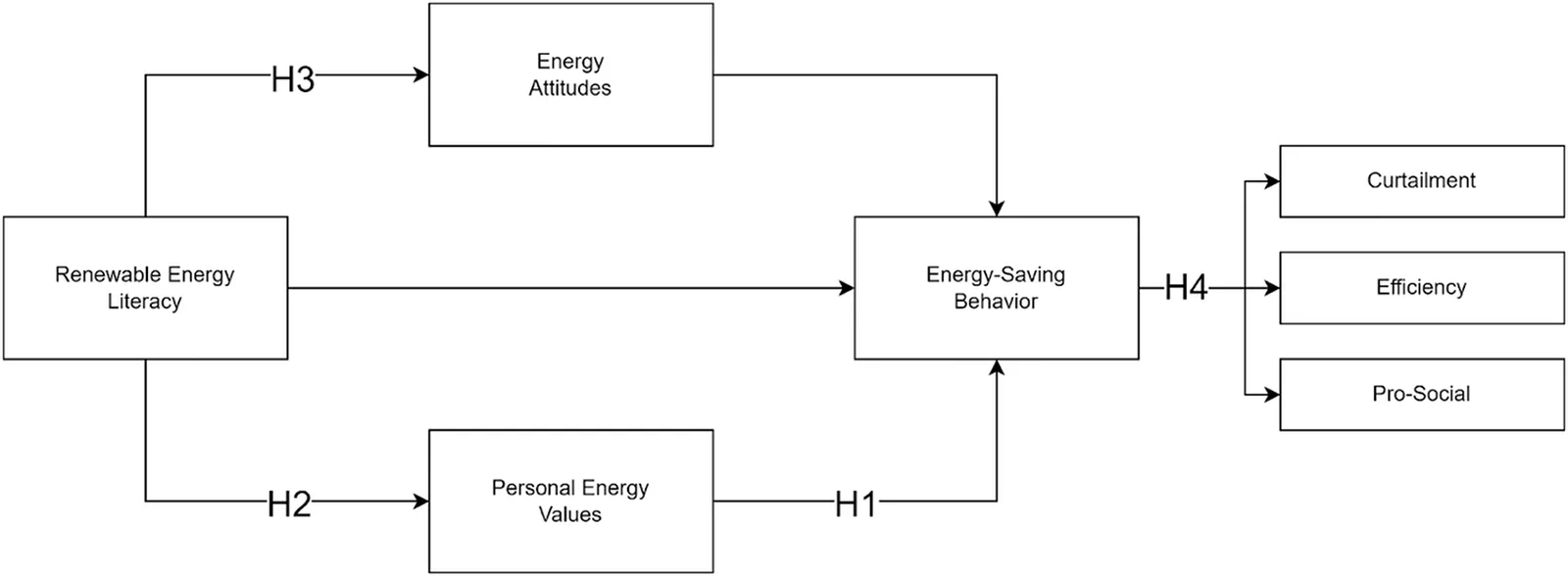
Conceptual framework illustrating direct and mediated pathways from renewable energy literacy to energy-saving behavior subdimensions through personal energy values and energy attitudes. REL → PEV → ESB (curtailment, efficiency, pro-social); REL → EA → ESB; REL → ESB (direct).

The conceptual framework is provided as Figure 1 in the Figure legends section and as a separate figure file for submission.

### Hypothesis development

**Renewableble energy literacy and energy-saving behavior**

Energy literacy has evolved from a predominantly technical construct to a multidimensional conception integrating cognitive knowledge, affective engagement, and behavioral disposition ( DeWaters & Powers, 2013). In the present study, REL is operationalized as the extent to which individuals possess substantive understanding of renewable energy technologies, their environmental and economic implications, and the practical means through which energy-saving practices may be incorporated into daily and professional life. From an energy economics perspective, this operationalization frames REL as energy-related human capital: the informational foundation that equips individuals to recognize conservation opportunities and make informed decisions about energy use consistent with both private interests and broader social objectives ( Mireku et al., 2023).

Empirical evidence supports a positive association between energy literacy and energy-saving behavior across diverse national and institutional contexts ( Lee et al., 2015; Santillan & Cedano, 2023; Pothitou et al., 2015). Household-level studies confirm that energy-related knowledge is associated with stronger conservation practices, even when socioeconomic factors are controlled ( Brounen et al., 2013), while Indonesian evidence documents positive associations between energy knowledge and conservation behavior among citizens ( Hendinata, Ardiwinata, & Pratama, 2022) and educational attainment groups ( Endriana et al., 2025). However, observed REL–ESB effect sizes are frequently modest, reflecting the operation of mediating behavioral mechanisms.

**Personal energy values as a behavioral mediator**

VBN theory ( Stern, 2000) posits that pro-environmental behavior emerges through a motivational sequence in which biospheric and altruistic value orientations shape ecological beliefs and personal norms, which in turn motivate behavioral commitment. In the energy domain, these value orientations constitute internalized conservation preferences that shape individuals’ energy-related decision-making beyond the immediate calculus of personal economic benefit ( Kim & Kim, 2024). From a behavioral perspective, the cultivation of energy values through REL may help orient individual energy decisions more consistently with conservation objectives, providing a channel through which literacy contributes to demand-side behavioral change ( do Paço & Varejo, 2010).

Research indicates that REL may contribute to the development of energy-relevant value orientations ( Akitsu & Ishihara, 2018). Evidence for the mediating role of energy values in the literacy–behavior relationship has been provided by Appiah et al. (2023), who demonstrated that energy-related values partially transmitted the influence of energy literacy on energy-saving behavior. Pothitou et al. (2015) similarly identified environmental predisposition (a construct closely analogous to personal energy values) as a significant predictor of energy attitudes and behaviors. Cross-national evidence further confirms robust associations between environmental value orientations and energy-saving behavior ( Kim & Kim, 2024; Al Mamun et al., 2022).

**Energy attitudes as a behavioral mediator**

The Theory of Planned Behavior ( Ajzen, 1991) identifies attitudes as important proximal determinants of behavioral intention and action. In the energy conservation context, favorable energy attitudes reflect individuals’ positive evaluations of conservation practices, including their perceptions of the economic rationality, environmental significance, and personal importance of energy saving. Pothitou et al. (2015) demonstrated that energy attitudes were significantly correlated with energy-saving habits and behaviors, while do Paço and Varejo (2010) showed that attitudinal orientations toward the environment distinguished energy-saving consumers from non-savers. Across diverse institutional and cultural contexts, favorable attitudes toward energy conservation are consistently associated with stronger behavioral engagement ( Puiu et al., 2025; Xuan et al., 2023), and meta-analytic evidence confirms the central importance of attitudinal factors in explaining variations in conservation behavior ( Carrus et al., 2021).

In the proposed mediation model, energy attitudes function as an evaluative bridge between REL and ESB. Individuals with higher REL are expected to form more favorable evaluative orientations toward energy conservation, insofar as enhanced understanding of the environmental and economic consequences of energy use renders conservation both instrumentally rational and normatively desirable. Empirical support for this attitudinal mediation pathway has been provided by Appiah et al. (2023) and by household energy studies demonstrating that awareness-related factors influence conservation outcomes partly through attitudinal processes ( Gajdzik et al., 2024; Zhao, Song, & Wang, 2019).

### Differential effects across energy-saving behavior subdimensions

Energy-saving behavior encompasses multiple distinct behavioral domains that differ substantially in their motivational requirements, structural conditions, and determinant profiles ( Frederiks, Stenner, & Hobman, 2015). Following Abrahamse and Steg (2009), the present study distinguishes among: curtailment behaviors (habitual reductions in energy use achieved through everyday behavioral adjustments); efficiency behaviors (adoption of energy-efficient technologies requiring capital investment); and pro-social behaviors (advocacy and collective influence promoting conservation within social and professional networks).

Curtailment and pro-social behaviors involve relatively low barriers to adoption, depending primarily on motivational readiness and attitudinal orientation rather than material resources ( Steg & Vlek, 2009). In contrast, efficiency behaviors typically require capital expenditure and structural access, conditions that depend on economic capacity rather than behavioral motivation alone ( Duy et al., 2023; Frederiks et al., 2015). This distinction implies that behavioral literacy programs may be most effective in promoting curtailment and pro-social behaviors, while efficiency behaviors may require complementary economic instruments to overcome structural barriers ( Stancu et al., 2025).

### Setting, participants, and data collection

The study population comprised members of the Indonesia Fuel Cell and Hydrogen Energy Association (IFHE), a professional body representing individuals with direct engagement in renewable energy systems, hydrogen technologies, and related energy sector activities. This population was selected on both theoretical and strategic grounds. Theoretically, IFHE members constitute an energy-literate professional community that provides appropriate variance in the mediating and outcome constructs while mitigating ceiling effects on the REL measure. From an energy economics perspective, energy sector professionals occupy a strategically significant position in Indonesia’s energy transition: their conservation behaviors, value orientations, and energy attitudes carry implications for organizational energy demand management and for the diffusion of conservation practices within professional networks.

Purposive sampling was employed to ensure the requisite domain knowledge among respondents. The study commenced and data collection began on 29 April 2026, after ethical approval had been obtained. Data were collected through a combination of online and in-person survey administration to maximize coverage within the IFHE membership. Before participating, all respondents received written information explaining the research purpose, the voluntary nature of participation, response anonymity, data confidentiality, and their right to decline or withdraw from the study. Written informed consent was obtained electronically from all participants by requiring them to select the consent button before accessing and completing the questionnaire. This consent procedure was included in and approved as part of the study’s ethics protocol. Following data screening for completeness and response quality, a final usable sample of 195 valid responses was obtained.

### Variables and measurement

All constructs were operationalized using a structured questionnaire with items rated on a four-point forced-choice Likert scale (1 = strongly disagree; 4 = strongly agree). The elimination of a neutral midpoint was designed to reduce acquiescence bias and encourage directional response.

Renewable Energy Literacy (REL) was measured using four items adapted from DeWaters and Powers (2013), reflecting respondents’ knowledge, understanding, and awareness of renewable energy systems and their implications for energy use and conservation, capturing cognitive dimensions including knowledge of energy sources, energy system functioning, and sustainability-related energy implications.

Personal Energy Values (PEV) were measured using four items capturing biospheric and altruistic value orientations relevant to energy use and environmental sustainability, grounded in VBN theory ( Stern, 2000), consistent with established operationalizations in the energy conservation literature ( Al Mamun et al., 2022; Appiah et al., 2023).

Energy Attitudes (EA) were assessed using three items reflecting respondents’ cognitive and affective evaluations of energy conservation practices, adapted from established TPB-based attitudinal instruments ( Ajzen, 1991; Puiu et al., 2025).

Energy-Saving Behavior (ESB) was operationalized as a multidimensional second-order construct comprising nine items across three first-order subdimensions: curtailment behavior (ESB_CUR; three items), efficiency behavior (ESB_EFF; three items), and pro-social energy action (ESB_PRO; three items), grounded in the taxonomy of Abrahamse and Steg (2009). The questionnaire underwent pilot testing prior to full deployment to verify semantic clarity, cultural appropriateness, and content validity.

### Statistical analysis

Data were analyzed using Partial Least Squares Structural Equation Modeling (PLS-SEM) implemented in SmartPLS 4. PLS-SEM was selected for its suitability for predictive and theory-testing research involving complex structural models, moderate sample sizes, non-normal data distributions, and higher-order construct specifications ( Hair, Hult, Ringle, & Sarstedt, 2017). The analysis proceeded through a two-stage sequential approach comprising measurement model assessment followed by structural model evaluation.

In the first stage, construct reliability was evaluated using Cronbach’s alpha (α ≥ .70) and composite reliability (CR ≥ .70). Convergent validity was assessed through standardized indicator loadings (λ ≥ .70) and Average Variance Extracted (AVE ≥ .50). Discriminant validity was examined using the Fornell–Larcker criterion and the heterotrait-monotrait ratio (HTMT < .90). The higher-order ESB construct was estimated using the disjoint two-stage approach ( Hair et al., 2017).

In the second stage, the structural model was evaluated through path coefficients (β), coefficients of determination (R ^2^), and effect sizes (f ^2^). Statistical significance of direct and indirect effects was assessed via bootstrapping with 5,000 resamples. Mediation effects were examined using the Variance Accounted For (VAF) index; VAF values between 20% and 80% indicate partial mediation, while values exceeding 80% indicate full mediation ( Hair et al., 2017).

### Preregistered data analysis

This study was not preregistered. The analysis plan was developed before the final statistical analysis was conducted, but it was not registered in an independent public registry.

## Results

### Respondent profile

Table 1 presents the demographic profile of respondents. The sample is predominantly male (n = 168, 86.2%), concentrated in the 25–34 age cohort (n = 91, 46.7%), and reflects the professional renewable energy community in Indonesia. Most respondents hold undergraduate (55.9%) or postgraduate (25.1%) qualifications. Household income is most frequently concentrated in the 5–10 million IDR range (50.8%), with 21.0% in the 10–20 million IDR range, consistent with the middle-income professional demographic of Indonesia’s urban energy sector. This income distribution is economically relevant: the predominance of middle-income respondents reflects economic constraints that may limit capital-intensive energy efficiency investment even among professionally energy-literate individuals.

**Table 1.** Demographic profile of respondents (N = 195).

Variable	Category	n	%
Gender	Male	168	86.2
Gender	Female	27	13.8
Age	25–34 years	91	46.7
Age	35–44 years	47	24.1
Age	45–54 years	30	15.4
Age	>55 years	13	6.7
Marital Status	Married	138	70.8
Marital Status	Unmarried	57	29.2
Education	High school	37	19.0
Education	Undergraduate	109	55.9
Education	Postgraduate	49	25.1
Household Income	< 5 million IDR	36	18.5
Household Income	5–10 million IDR	99	50.8
Household Income	10–20 million IDR	41	21.0
Household Income	> 20 million IDR	8	4.1
Household Income	Prefer not to disclose	11	5.6

### Measurement model assessment

Table 2 presents the measurement model results. The majority of indicator loadings exceed the recommended threshold of.70, affirming acceptable indicator reliability. Three indicators (REL2 = 0.694, REL3 = 0.693, VAL3 = 0.694) produced loadings marginally below.70; these were retained given their substantive contribution to construct coverage and content validity. Cronbach’s alpha values range from.602 (ESB_CUR) to.839 (ESB_EFF), and composite reliability estimates exceed.70 for most constructs, affirming adequate internal consistency. AVE values meet or approach the.50 threshold for all constructs, supporting convergent validity.

**Table 2.** Measurement model results.

Construct	Indicator	Loading	Cronbach’s α	Comp. Reliability	AVE
Energy Attitudes (EA)	ATT1	0.802	0.628	0.647	0.577
Energy Attitudes (EA)	ATT2	0.641			
Energy Attitudes (EA)	ATT3	0.823			
ESB — Curtailment (ESB_CUR)	CUR1	0.760	0.602	0.790	0.556
ESB — Curtailment (ESB_CUR)	CUR2	0.719			
ESB — Curtailment (ESB_CUR)	CUR3	0.758			
ESB — Efficiency (ESB_EFF)	EFF1	0.876	0.839	0.903	0.757
ESB — Efficiency (ESB_EFF)	EFF2	0.831			
ESB — Efficiency (ESB_EFF)	EFF3	0.901			
ESB — Pro-Social (ESB_PRO)	PRO1	0.851	0.704	0.835	0.629
ESB — Pro-Social (ESB_PRO)	PRO2	0.786			
ESB — Pro-Social (ESB_PRO)	PRO3	0.740			
Renewable Energy Literacy (REL)	REL1	0.791	0.740	0.837	0.563
Renewable Energy Literacy (REL)	REL2	0.694			
Renewable Energy Literacy (REL)	REL3	0.693			
Renewable Energy Literacy (REL)	REL4	0.815			
Personal Energy Values (PEV)	VAL1	0.834	0.785	0.862	0.611
Personal Energy Values (PEV)	VAL2	0.780			
Personal Energy Values (PEV)	VAL3	0.694			
Personal Energy Values (PEV)	VAL4	0.811			

*Note:* Loadings below.70 were retained based on substantive contribution to construct coverage. Cronbach’s alpha for ESB_CUR (α = .602) and EA (α = .628) fall below the conventional.70 threshold; however, composite reliability and AVE values for all constructs meet established criteria ( Hair et al., 2017), justifying retention of these measurement specifications. Readers should note that the composite reliability of EA (CR = 0.647) is also marginally below the conventional threshold; the substantive interpretation of EA-mediated findings should be considered in light of this measurement limitation.

Table 3 presents discriminant validity results using the Fornell–Larcker criterion. The square root of the AVE for each construct (diagonal values in bold) exceeds its correlations with all other constructs, confirming the empirical distinctiveness of each measurement instrument. Heterotrait-monotrait ratio (HTMT) values were below the.90 threshold for all construct pairs, providing additional confirmation of satisfactory discriminant validity.

**Table 3.** Discriminant validity — Fornell–Larcker criterion.

Construct	EA	ESB	ESB_CUR	ESB_EFF	ESB_PRO	REL	PEV
EA	0.759						
ESB	0.624	0.621					
ESB_CUR	0.639	0.806	0.746				
ESB_EFF	0.345	0.682	0.289	0.870			
ESB_PRO	0.473	0.845	0.594	0.342	0.793		
REL	0.574	0.599	0.579	0.230	0.573	0.750	
PEV	0.517	0.677	0.674	0.352	0.549	0.586	0.781

*Note:* Diagonal values (bold) represent the square root of AVE; off-diagonal values are inter-construct correlations. EA = Energy Attitudes; ESB = Energy-Saving Behavior; REL = Renewable Energy Literacy; PEV = Personal Energy Values.

**Figure 2.**
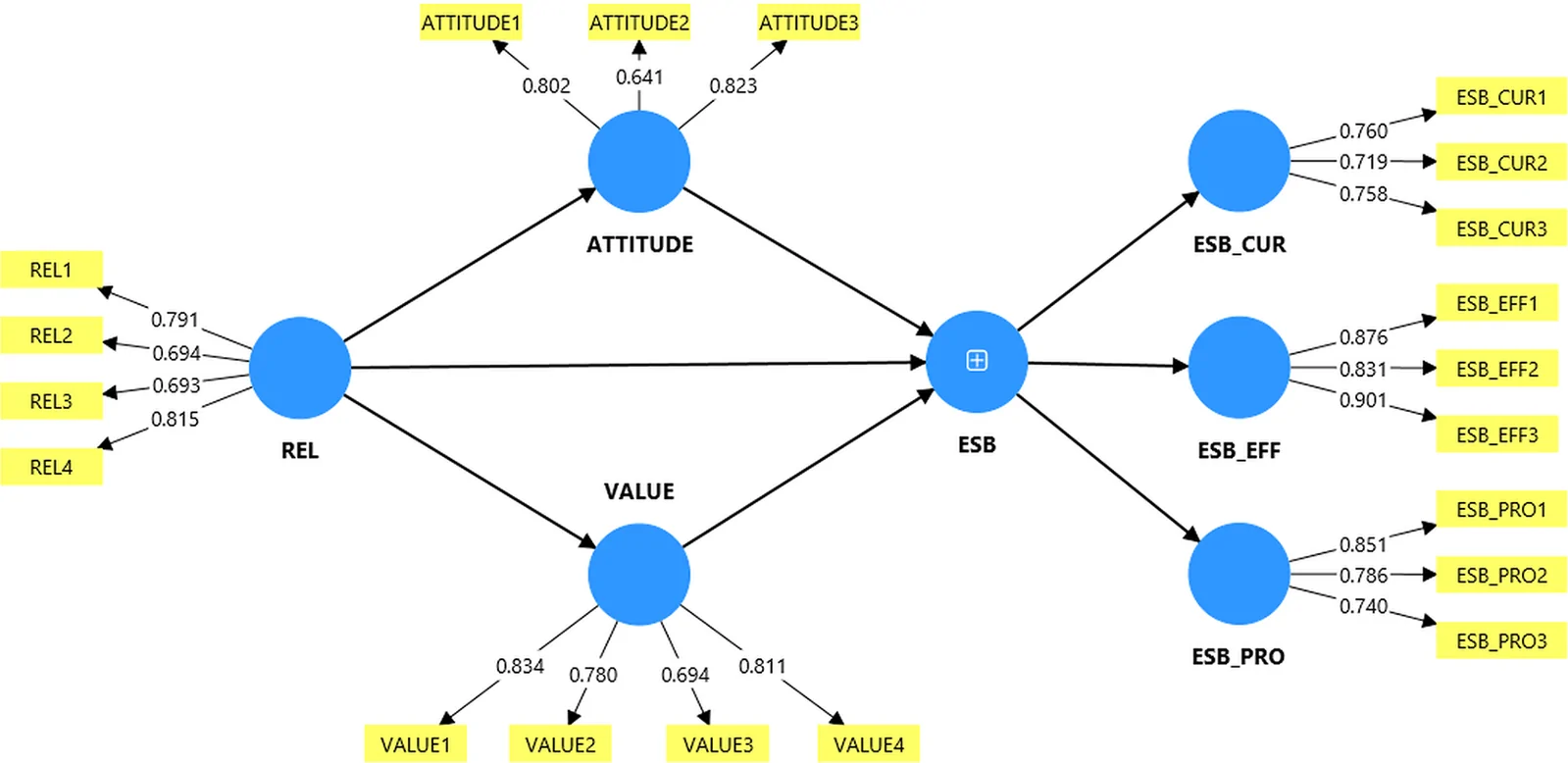
Measurement model diagram (outer loadings).

The measurement model diagram is provided as Figure 2 in the Figure legends section and as a separate figure file for submission.

### Structural model assessment

Table 4 presents the structural model results. REL is a significant and substantial predictor of both PEV (β = 0.586, t = 10.238, p < .001, f ^2^ = 0.522, large effect) and EA (β = 0.574, t = 9.915, p < .001, f ^2^ = 0.491, medium effect), confirming that renewable energy literacy strongly determines both the value orientations and attitudinal orientations of IFHE members toward energy conservation. Both mediating constructs significantly influence ESB: PEV demonstrates a medium effect (β = 0.411, t = 5.685, p < .001, f ^2^ = 0.244), while EA shows a smaller but significant effect (β = 0.307, t = 4.306, p < .001, f ^2^ = 0.139). The direct effect of REL on ESB is statistically significant but modest (β = 0.182, t = 2.273, p = .023, f ^2^ = 0.044), consistent with the presence of substantial mediation. The model accounts for 57.9% of the variance in ESB (R ^2^ = 0.579).

**Table 4.** Structural model results.

Path	β	t-value	p-value	R ^2^	f ^2^	Effect Size
REL → EA	0.574	9.915	<.001	0.329	0.491	Medium
REL → PEV	0.586	10.238	<.001	0.343	0.522	Large
REL → ESB (direct)	0.182	2.273	.023	0.579	0.044	Small
EA → ESB	0.307	4.306	<.001	—	0.139	Small
PEV → ESB	0.411	5.685	<.001	—	0.244	Medium
ESB → ESB_CUR	0.806	33.531	<.001	0.650	1.859	Large
ESB → ESB_EFF	0.682	7.894	<.001	0.465	0.870	Large
ESB → ESB_PRO	0.845	33.803	<.001	0.714	2.497	Large

*Note:* REL = Renewable Energy Literacy; EA = Energy Attitudes; PEV = Personal Energy Values; ESB = Energy-Saving Behavior; β = standardized path coefficient; β ^2^ = effect size; R ^2^ = coefficient of determination.

**Figure 3.**
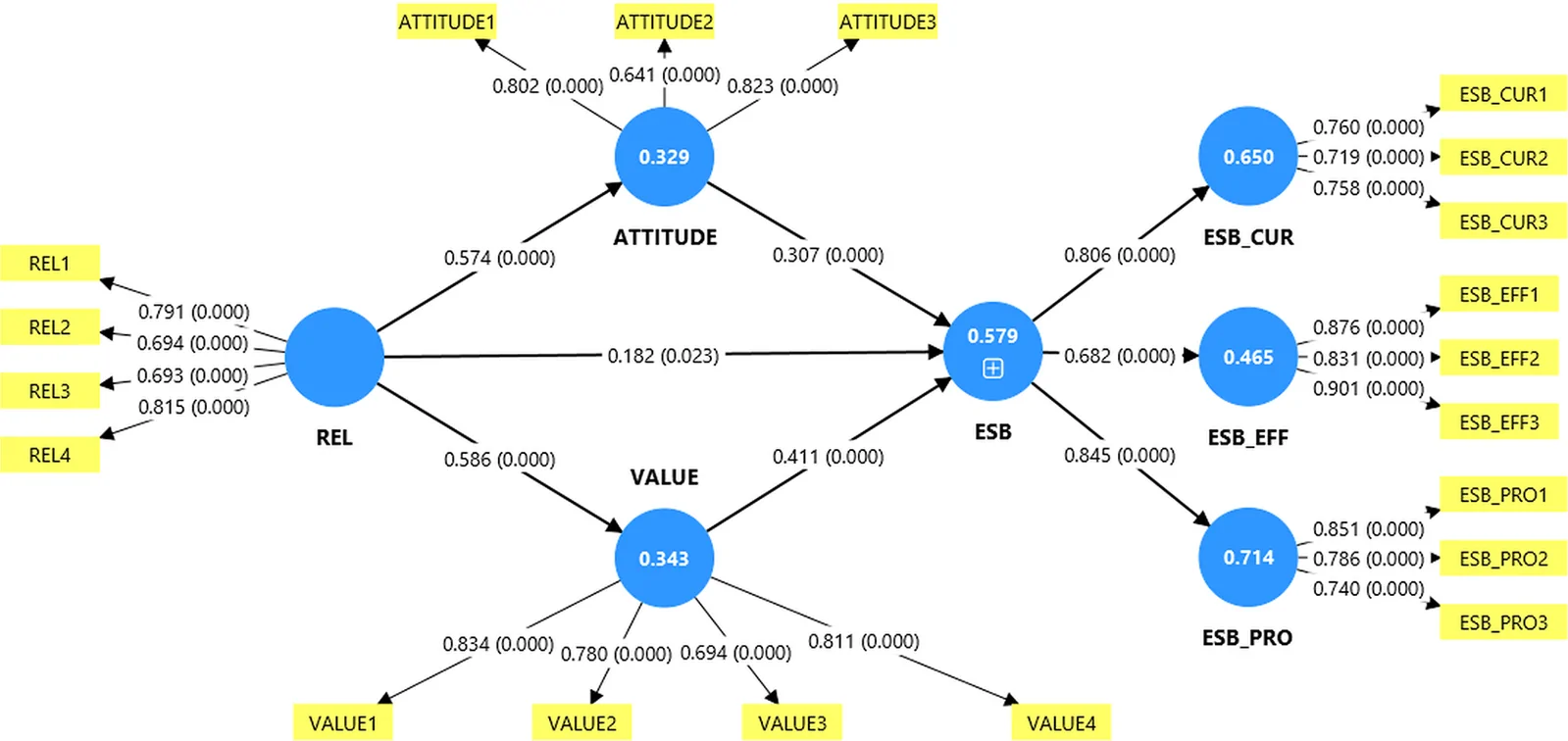
Structural model.

The structural model is provided as Figure 3 in the Figure legends section and as a separate figure file for submission.

### Hypothesis testing and mediation analysis

Table 5 presents the mediation analysis results. The total effect of REL on ESB is significant and substantial (β = 0.599, t = 11.371, p < .001). The direct effect accounts for approximately 30% of the total REL–ESB relationship (β = 0.182), while the indirect effects through PEV (β = 0.241, VAF = 57%) and EA (β = 0.176, VAF = 49%) together account for approximately 70% of the total effect (VAF = 69.6%), consistent with partial mediation through both pathways. H1, H2, and H3 are all supported.

**Table 5.** Mediation analysis results.

Path	Direct β	Indirect β	Total β	t-value	p-value	VAF (%)	Mediation
REL → ESB	0.182	0.417	0.599	11.371	<.001	69.6%	Partial
REL → PEV → ESB	—	0.241	—	5.008	<.001	57%	Partial
REL → EA → ESB	—	0.176	—	4.681	<.001	49%	Partial
PEV → ESB	0.411	—	0.411	5.685	<.001	—	Direct
EA → ESB	0.307	—	0.307	4.306	<.001	—	Direct

*Note:* VAF = Variance Accounted For. REL = Renewable Energy Literacy; PEV = Personal Energy Values; EA = Energy Attitudes; ESB = Energy-Saving Behavior.

Supporting H4, subdimensional mediation analysis revealed that indirect REL effects through the behavioral mediators were strongest for pro-social behavior (β = 0.259) and curtailment behavior (β = 0.247), with comparatively weaker mediated effects for efficiency behavior (β = 0.209), confirming that literacy-driven behavioral pathways are differentially effective across ESB subdimensions.

## Discussion

### Renewable energy literacy as a demand-side behavioral determinant

The finding that REL exerts a statistically significant but modest direct effect on ESB (β = 0.182, p = .023, f ^2^ = 0.044) supports H1 and provides evidence that renewable energy literacy functions as a behavioral determinant of energy conservation, while also indicating the limitations of knowledge provision as a standalone demand-side policy instrument. From a behavioral energy economics perspective, this finding positions REL as energy-related human capital: the cognitive prerequisite for informed energy conservation decisions, analogous to the role of financial literacy in household financial behavior ( Mireku et al., 2023). Just as financial literacy creates the informational conditions for sound financial decisions without guaranteeing optimal behavioral outcomes, energy literacy equips individuals with the cognitive foundation for conservation while requiring complementary motivational and evaluative processes to generate actual behavioral change.

This interpretation is consistent with substantial prior evidence on the knowledge–behavior gap. do Paço and Varejo (2010) demonstrated that even among environmentally concerned consumers, energy knowledge did not reliably distinguish energy savers from non-savers; rather, it was the economic cost of electricity that most powerfully differentiated these behavioral groups, suggesting that knowledge becomes behaviorally consequential primarily when accompanied by economically relevant evaluations and motivations. Pothitou et al. (2015) similarly found that the path from knowledge to actual conservation behavior was considerably weaker than the attitude–behavior pathway, a pattern directly mirrored in the present study’s finding that REL’s indirect effects through PEV and EA (β = 0.417) substantially exceed the direct effect (β = 0.182).

The relatively small direct effect size (f ^2^ = 0.044) has an implication for Indonesia’s demand-side energy management agenda: energy literacy campaigns that deliver knowledge without simultaneously cultivating conservation values and favorable attitudes are unlikely to generate the sustained behavioral change needed to contribute meaningfully to household energy demand reduction. The significant residual direct effect (β = 0.182, p = .023) nevertheless indicates that REL makes a behavioral contribution beyond what is captured by the proposed mediators, reflecting proximal cognitive processes (including enhanced awareness of energy-saving opportunities and increased behavioral self-efficacy) that operate independently of value and attitudinal channels.

### Personal energy values as a demand-side behavioral mechanism

The substantial mediation of the REL–ESB relationship by personal energy values (β = 0.241, VAF = 57%) supports H2 and suggests that values-driven processes constitute an important behavioral mechanism through which energy literacy influences conservation outcomes. The alignment of energy literacy with conservation values may help orient individual energy decisions more consistently with broader environmental and social objectives, suggesting a potential channel through which literacy contributes to demand-side behavioral change.

The magnitude of the PEV-mediation pathway (VAF = 57%) indicates that more than half of REL’s total behavioral effect operates through the cultivation of conservation-oriented values. This finding extends the prior evidence of Appiah et al. (2023) and is consistent with Pothitou et al.’s (2015) demonstration that environmental predisposition served as an important intermediary between environmental knowledge and conservation behavior. The cross-national evidence of Kim and Kim (2024) further confirms robust positive associations between environmental value orientations and energy-saving behavior across diverse institutional settings.

An economically significant implication concerns the potential durability of literacy-driven conservation outcomes. Prior research suggests that values-based motivations may be relatively stable across varying economic conditions ( do Paço & Varejo, 2010). If so, individuals with stronger conservation values cultivated through REL may maintain conservation behavior more consistently than those motivated primarily by economic factors. The present cross-sectional data do not directly test this longitudinal inference, but the findings are consistent with this interpretation.

### Energy attitudes as an evaluative pathway for conservation behavior

The significant mediation of the REL–ESB relationship through energy attitudes (β = 0.176, VAF = 49%) provides evidence for a second, evaluative pathway through which REL may influence conservation outcomes, consistent with TPB’s identification of attitudes as important proximal determinants of behavioral intention and action ( Ajzen, 1991). The attitudinal mediation pathway accounts for approximately half of the total REL–ESB relationship, suggesting the policy significance of attitude formation as a demand-side behavioral process.

The finding that the EA-mediation pathway (VAF = 49%) is somewhat smaller than the PEV-mediation pathway (VAF = 57%) reflects the theoretical distinction between the roles of values and attitudes as behavioral determinants. Personal energy values represent stable, broad motivational orientations; energy attitudes reflect more context-specific evaluative judgments that may be more responsive to shorter-term behavioral interventions (including economic framing of energy-saving decisions and social comparison messages). This distinction carries complementary policy implications: attitudinal interventions may be deployed as shorter-term complements to the deeper value cultivation strategies implied by the PEV-mediation finding.

### Differential conservation behavior domains and policy differentiation

This finding contributes to research on behavioral dimensions of energy conservation and demand-side energy management. While energy-saving behavior is frequently conceptualized as a homogeneous outcome, the present results suggest that renewable energy literacy does not influence all conservation behaviors uniformly. By distinguishing among curtailment, efficiency, and pro-social behaviors, this study reveals that literacy-driven behavioral mechanisms operate differently across conservation domains. The findings suggest that the assumption of behavioral homogeneity may overlook important differences across conservation domains.

The subdimensional analysis provides partial support for H4, demonstrating that the behavioral pathways through which REL influences conservation are differentially effective across domains. The mediated effects of REL are strongest for pro-social behavior (β = 0.259) and curtailment behavior (β = 0.247), with comparatively weaker effects for efficiency behavior (β = 0.209). This differential pattern has important implications for the design and targeting of demand-side energy management instruments.

The stronger mediated effects for curtailment and pro-social behaviors are consistent with the theoretical expectation that these domains are primarily constrained by motivational and attitudinal factors rather than structural and economic barriers. Curtailment behaviors involve low-cost adjustments within individuals’ direct behavioral control, with primary barriers being motivational and attitudinal. For these domains, literacy programs capable of strengthening conservation values and favorable energy attitudes represent appropriate and potentially cost-effective demand-side instruments.

The comparatively weaker mediated effects for efficiency behavior (β = 0.209) are consistent with the structural barriers highlighted in the energy efficiency literature ( Frederiks et al., 2015). Efficiency behaviors require capital expenditure and access to energy-efficient technology that behavioral motivation and attitudinal change alone cannot generate. This pattern is consistent with the evidence reported by Duy et al. (2023), who demonstrated that household adoption of micro-renewable energy systems in Vietnam was significantly constrained by income, educational, and geographic factors independent of behavioral motivations. In the Indonesian context, where middle-income household budgets remain constrained and access to energy-efficient technologies varies substantially, the weaker efficiency behavior response to literacy-driven mediation is economically explicable ( Stancu et al., 2025).

These findings suggest that demand-side strategies targeting efficiency behavior would benefit from complementing behavioral literacy programs with economic instruments that address structural barriers, including appliance efficiency subsidies, tax incentives for energy-efficient capital investment, minimum efficiency standards, and green procurement mandates. This complementary policy architecture represents an evidence-based approach to demand-side management supported by the subdimensional findings of the present study.

### Policy implications for demand-side energy management

Three principal policy implications follow from the empirical results. First, energy literacy programs should be explicitly positioned as demand-side energy management instruments within Indonesia’s energy transition strategy. The total effect of REL on ESB (β = 0.599) suggests that investments in renewable energy literacy may generate meaningful conservation outcomes when programs cultivate conservation values and favorable attitudes alongside cognitive knowledge. Indonesia’s Ministry of Energy and Mineral Resources, along with professional associations such as IFHE, should integrate behavioral objectives into energy literacy program design, moving beyond technical information dissemination toward holistic behavioral interventions. This repositioning of energy literacy programs as demand-side management tools would align Indonesia’s behavioral conservation strategy with supply-side renewable energy investments currently receiving policy attention ( IEA, 2023; Yildiz, 2026).

Second, the dual-pathway finding, in which PEV (VAF = 57%) and EA (VAF = 49%) jointly account for approximately 70% of the total REL–ESB relationship, implies that demand-side programs should target both behavioral pathways simultaneously. Values-based interventions delivered through sustained professional community engagement can cultivate stable motivational orientations generating durable conservation behavior. Attitude-based interventions, including economic framing of conservation benefits and social comparison programs, can shift evaluative orientations more rapidly and may produce shorter-term behavioral returns.

Third, the comparatively weaker response observed for efficiency-related behaviors suggests the need for a complementary economic policy architecture alongside behavioral programs. For efficiency-related behaviors, the findings are consistent with the possibility that financial and structural barriers limit the effectiveness of literacy-driven interventions for efficiency-related behaviors. Economic instruments including appliance efficiency subsidies, tax credits for energy-efficient investments, low-interest green financing for energy-sector professionals, and minimum efficiency standards are essential complements in the Indonesian context ( Duy et al., 2023; IEA, 2023; Hardi et al., 2024).

## Conclusions

This study examined the direct and mediated effects of renewable energy literacy on multidimensional energy-saving behavior among members of the Indonesia Fuel Cell and Hydrogen Energy Association using an integrated VBN–TPB framework. The findings suggest that renewable energy literacy is associated with energy-saving behavior both directly and through personal energy values and energy attitudes as partial mediators. The results further indicate that literacy-driven behavioral pathways are more effective for curtailment and pro-social behaviors than for efficiency-related behaviors.

These findings suggest that renewable energy literacy contributes to conservation behavior not only by increasing knowledge but also by strengthening conservation-oriented values and favorable energy attitudes. More importantly, the effectiveness of literacy-driven mechanisms varies across behavioral domains, indicating that energy-saving behavior should not be treated as a homogeneous construct. While literacy appears effective in promoting low-cost and socially oriented conservation actions, efficiency-related behaviors remain influenced by economic and structural constraints that extend beyond knowledge and motivation alone.

From a policy perspective, the findings support the integration of behavioral interventions into demand-side energy management strategies. Programs aimed at improving renewable energy literacy should be designed to cultivate both conservation values and positive energy attitudes. However, literacy-based interventions alone are unlikely to be sufficient for promoting efficiency-related behaviors, which require complementary economic instruments and structural support.

Several limitations should be acknowledged. The cross-sectional design limits causal inference, the professional composition of the sample constrains generalizability, and the use of self-reported measures introduces the possibility of response bias. Future research should employ longitudinal designs, objective energy consumption indicators, and economic variables such as energy expenditure, price sensitivity, and income to further advance understanding of literacy-driven conservation behavior in energy transition contexts.

## Ethical considerations

This study was conducted in accordance with ethical principles for research involving human participants. The study protocol was approved by Universitas Sebelas Maret, approval number 11729/UN27.02/PT.01.04/2026, on 27 April 2026. The study commenced on 29 April 2026, after ethical approval had been obtained. Before participating, all respondents received written information explaining the research purpose, the voluntary nature of participation, response anonymity, data confidentiality, and their right to decline or withdraw from the study. Written informed consent was obtained electronically from all participants by requiring them to select the consent button before accessing and completing the questionnaire. This consent procedure was included in and approved as part of the study’s ethics protocol. No personal identifiers were collected or retained in the analytical dataset.

## Use of artificial intelligence (AI)

Generative AI tools were used to assist with language editing, drafting, and structural refinement of the manuscript. No AI tools were used for data collection, statistical analysis, interpretation of results, or generation of research findings. All intellectual content, analysis, and conclusions represent the authors’ own work and responsibility.

## Data Availability

The individual-level survey data underlying the results of this study are not publicly available under an open licence because respondents consented to participation in this study and to restricted academic or verification access only; the consent process did not include unrestricted public deposition or public reuse of participant-level survey data. Public release could create a risk of deductive disclosure because the dataset was obtained from a defined professional community, the Indonesia Fuel Cell and Hydrogen Energy Association (IFHE), and includes demographic and professional characteristics that may be identifiable in small subgroups. The restriction therefore applies to public deposition and unrestricted reuse of participant-level data; no direct personal identifiers were collected or retained in the analytical dataset. The Universitas Sebelas Maret ethics approval did not authorize unrestricted public sharing of participant-level survey data. To support transparency and verification, de-identified intermediary materials that do not compromise participant anonymity may be made available on reasonable request, including aggregate descriptive outputs, the anonymized variable dictionary/codebook, the questionnaire and measurement items, and the SmartPLS output used to support the reported tables. A de-identified participant-level dataset may be considered for access only where the request is for reasonable academic or verification purposes and where disclosure does not compromise participant anonymity. The extended materials supporting this study, including the survey questionnaire, measurement items, data dictionary/codebook, SmartPLS output, and reporting checklist, are not deposited publicly because these materials are linked to the restricted survey dataset and study documentation. Extended materials that can be shared without compromising anonymity may be requested from the corresponding author for reasonable academic and verification purposes, subject to the confidentiality and data-use conditions described below. Requests for underlying data or extended materials should be sent to the corresponding author, Dewi Kusuma Wardani ( dewikusuma@staff.uns.ac.id). Requests should include the requester’s name, institutional affiliation, research purpose, proposed analysis plan, exact data or materials requested, ethics approval or exemption where applicable, and a statement confirming that the requester will not attempt to re-identify participants, redistribute the data or materials, or use them for purposes other than those approved by the authors. Access will be considered by the corresponding author and research team and may be granted subject to ethical, privacy, confidentiality, and institutional requirements, including institutional or ethics approval where required and a signed data-use or confidentiality agreement. Data analysis was conducted using SmartPLS 4. No custom software or original programming code was developed for this study. The analysis used standard SmartPLS procedures for measurement model assessment, structural model assessment, bootstrapping, and mediation analysis. SmartPLS project files and statistical output files may be requested from the corresponding author for reasonable academic and verification purposes, subject to the confidentiality and data-use conditions described in the Data availability section. This cross-sectional survey study was reported in accordance with the STROBE reporting guideline for observational studies. The completed STROBE checklist may be requested from the corresponding author for reasonable academic and verification purposes, subject to the conditions described in the Data availability section.
