# Renewable Energy Literacy and Multidimensional Energy-Saving Behavior in Indonesia: The Roles of Personal Energy Values and Energy Attitudes

**DOI:** 10.12688/f1000research.185821.2

**Published:** 2026-11-09

**Authors:** Rahmadi Budiman, Dewi Kusuma Wardani, Aniek Hindrayani, Muhammad Sabandi

**Affiliations:** 1 Department of Economics Education, Faculty of Teacher Training and Education, Sebelas Maret University, Surakarta, Central Java, Indonesia

**Keywords:** renewable energy literacy; energy-saving behavior; energy demand management; energy transition; behavioral determinants; PLS-SEM; Indonesia

## Abstract

**Background:**

Demand-side behavioral change is an important component of sustainable energy transitions, yet limited evidence explains how renewable energy literacy translates into distinct forms of energy-saving behavior in emerging economies.

**Methods:**

This cross-sectional quantitative explanatory study surveyed 195 members of the Indonesia Fuel Cell and Hydrogen Energy Association. Renewable energy literacy was modeled as the predictor, personal energy values and energy attitudes as mediators, and energy-saving behavior as a multidimensional second-order construct comprising curtailment, efficiency, and pro-social behaviors. Data were analyzed using Partial Least Squares Structural Equation Modeling in SmartPLS 4 with 5,000 bootstrap resamples.

**Results:**

Renewable energy literacy was positively associated with energy-saving behavior directly and indirectly through personal energy values and energy attitudes. The total association between renewable energy literacy and energy-saving behavior was substantial (β = 0.599, p < .001), while the direct association was smaller (β = 0.182, p = .023). Significant indirect associations were observed through personal energy values (β = 0.241, p < .001) and energy attitudes (β = 0.176, p < .001), consistent with partial mediation within the specified model.

**Conclusions:**

Within this professional renewable energy sample, renewable energy literacy was positively associated with energy-saving behavior, with significant indirect associations through personal energy values and energy attitudes. These findings highlight the relevance of value and attitudinal correlates in understanding energy-saving behavior among energy-sector professionals. Because the study is cross-sectional and based on a specialized professional sample, the results should not be interpreted as evidence of causal mechanisms or generalized directly to the Indonesian public.

## Introduction

The global transition toward renewable energy systems requires coordinated action on both the supply and demand sides of energy markets. While governments, international institutions, and the private sector have directed substantial resources toward expanding renewable energy infrastructure and reforming energy policy frameworks, the ultimate effectiveness of these supply-side measures depends critically on whether individual and organizational energy users adopt conservation behaviors that reduce aggregate demand ( Yildiz, 2026; IEA, 2023). Behavioral change at the household and organizational level is increasingly recognized as an important complement to supply-side investment in achieving energy transition objectives. The importance of demand-side management is further supported by evidence that supply-side renewable energy expansion alone is insufficient to meet emissions reduction commitments; sustained reductions in energy consumption intensity are required alongside the decarbonization of energy supply ( Yildiz, 2026; Berrich, Mafakheri, & Dabbou, 2024).

Household and organizational energy consumption constitute particularly significant domains for demand-side management. In the Asia-Pacific region, rapidly developing economies such as Vietnam and Indonesia are experiencing pronounced growth in residential and commercial energy consumption, driven by rising incomes, accelerating urbanization, and increased ownership of energy-consuming appliances and technologies. Duy, Cassells, and Hanly (2023) demonstrated that household electricity consumption in Vietnam grew by over 20% in just two years, driven by income growth and lifestyle change, and that behavioral and structural interventions (including household adoption of micro-renewable energy systems) can generate meaningful reductions in residential energy expenditure. This evidence underscores the economic significance of demand-side behavioral change as a complement to renewable energy supply expansion in emerging economies ( Duy et al., 2023). In Indonesia, these dynamics are compounded by the country’s ambitious net-zero commitments and the structural challenge of transitioning an economy that remains heavily dependent on fossil fuel-based energy ( IEA, 2023; Hardi et al., 2024).

Among the behavioral determinants of household and professional energy use that have attracted growing scholarly attention, renewable energy literacy (REL) occupies an increasingly prominent position. From an energy economics perspective, REL represents a form of energy-related human capital: the knowledge, understanding, and competencies that equip individuals with the informational basis for informed energy decision-making and conservation behavior ( DeWaters & Powers, 2013). This framing parallels the established relationship between financial literacy and household financial behavior: just as financially literate individuals are better positioned to make informed savings and investment decisions ( Mireku, Appiah, & Agana, 2023), energy-literate individuals are better equipped to identify and act upon energy conservation opportunities in their professional and household lives. Empirical evidence consistently documents that higher levels of energy literacy are positively associated with conservation-oriented behaviors across diverse national and institutional contexts ( Lee, Lee, Altschuld, & Pan, 2015; Pothitou, Varga, Kolios, & Gu, 2015; Santillan & Cedano, 2023).

However, a persistent knowledge–behavior gap presents a practical challenge for demand-side energy management policy: individuals possessing adequate energy knowledge do not always translate such understanding into corresponding conservation practices, particularly at the household level where energy costs, technology access, and behavioral norms interact to shape energy decisions ( Brounen, Kok, & Quigley, 2013; do Paço & Varejo, 2010). Pothitou et al. (2015) demonstrated that while environmental knowledge and predisposition were significantly correlated with energy attitudes, habits, and behaviors in their household study, the association between knowledge and actual conservation behavior was considerably attenuated relative to the attitude–behavior link. do Paço and Varejo (2010), in their analysis of energy-saving behavior among Portuguese consumers, found that even among environmentally concerned respondents, economic factors (particularly electricity cost) constituted the primary differentiator between energy savers and non-savers. These findings suggest that information provision alone is insufficient to close the behavioral gap in energy demand management, and that motivational and attitudinal mechanisms must be understood and targeted to generate effective conservation outcomes.

Two theoretical frameworks provide complementary but partial lenses for interpreting these behavioral mechanisms. Value–Belief–Norm (VBN) theory ( Stern, 2000) positions value orientations upstream of ecological beliefs and personal norms, which in turn contribute to pro-environmental behavioral commitment ( Al Mamun et al., 2022; Kim & Kim, 2024). The Theory of Planned Behavior (TPB; Ajzen, 1991) conceptualizes attitudes as one component of a broader intention-based framework that also includes subjective norms and perceived behavioral control. The present study does not attempt to operationalize or test either theory in its complete form. Instead, it draws selectively on the value component of VBN to interpret personal energy values and on the attitudinal component of TPB to interpret energy attitudes. Ecological beliefs, personal norms, subjective norms, perceived behavioral control, and behavioral intention are not included in the present model. Accordingly, VBN and TPB are used as complementary theoretical lenses for interpreting two selected psychological correlates of energy-saving behavior rather than as fully specified theoretical models ( Puiu et al., 2025; Xuan et al., 2023).

These issues are particularly salient in the Indonesian context. Indonesia is simultaneously one of Southeast Asia’s largest and most rapidly growing economies and one of the most critical emerging energy markets for global climate policy. The country has committed to achieving net-zero emissions by 2060 and is navigating the complex economic and institutional reforms required to shift from fossil fuel dependence toward sustainable energy systems ( IEA, 2023; IESR, 2025). In this context, understanding the behavioral factors that encourage energy conservation is increasingly important, as demand-side actions constitute a critical complement to technological innovation and energy policy interventions.

Despite growing evidence that renewable energy literacy promotes conservation-oriented behavior, an important theoretical and policy question remains unresolved: how does energy literacy translate into actual conservation action, particularly when conservation behaviors differ in their economic requirements and structural constraints? Existing research consistently reports positive associations between literacy and energy-saving behavior, yet the observed effects are often modest, suggesting that knowledge alone is insufficient to generate sustained behavioral change. This persistent knowledge–behavior gap indicates the need to examine the motivational and evaluative mechanisms through which literacy influences conservation decisions.

While Appiah et al. (2023) demonstrated that energy-related values and attitudes partially mediate the relationship between energy literacy and energy-saving behavior, two important questions remain unaddressed. First, their study treats energy-saving behavior as a composite outcome, which precludes examination of whether literacy-driven pathways operate with equal strength across behaviorally distinct conservation domains. Second, evidence from professional energy communities in emerging economies undergoing active energy transition (a context that differs substantially from student and general household samples) remains limited. The present study addresses both gaps by operationalizing energy-saving behavior as a multidimensional second-order construct comprising curtailment, efficiency, and pro-social behavioral subdimensions, and by drawing on a professional renewable energy community in Indonesia as its study population.

Furthermore, energy-saving behavior has frequently been treated as a homogeneous construct despite encompassing multiple behavioral domains with distinct economic and behavioral characteristics. Curtailment behaviors typically require routine behavioral adjustments, efficiency behaviors often involve financial investment and technological adoption, whereas pro-social behaviors depend on social engagement and advocacy. Consequently, the pathways linking renewable energy literacy to these behavioral domains may not operate uniformly. Understanding such differences is particularly important in emerging economies undergoing energy transition, where economic constraints and institutional conditions may shape the effectiveness of literacy-based conservation interventions.

To address these gaps, this study examines the direct and indirect associations between renewable energy literacy and multidimensional energy-saving behavior through personal energy values and energy attitudes. Rather than testing complete VBN and TPB models, the study draws selectively on VBN to interpret the value-oriented component represented by PEV and on TPB to interpret the evaluative component represented by EA. This theory-informed framework allows both constructs to be examined simultaneously while retaining the multidimensional specification of energy-saving behavior.

Building on these gaps, the present study pursues two interconnected research objectives: (a) to determine whether REL is positively associated with multidimensional energy-saving behavior; and (b) to assess whether PEV and EA statistically mediate the REL–ESB association within the specified cross-sectional model. Three hypotheses are advanced:

H1: Renewable energy literacy is positively associated with energy-saving behavior.

H2: Renewable energy literacy is positively associated with energy-saving behavior indirectly through personal energy values.

H3: Renewable energy literacy is positively associated with energy-saving behavior indirectly through energy attitudes.

This study contributes to the behavioral energy economics and energy conservation literature in three interconnected ways. First, it extends prior research by examining whether renewable energy literacy exerts differential effects across distinct behavioral domains of energy-saving behavior. By operationalizing ESB as a multidimensional second-order construct comprising curtailment, efficiency, and pro-social subdimensions, the study moves beyond the common treatment of ESB as a homogeneous outcome and provides evidence on whether literacy-driven pathways are equally effective across these domains. Second, it draws selectively on VBN theory and TPB within a unified PLS-SEM framework, using personal energy values as a value-oriented construct informed by VBN and energy attitudes as an attitudinal construct informed by TPB. The framework is not intended as a complete empirical test of either theory.

## Methods

### Study design and conceptual framework

This study employs a cross-sectional, quantitative explanatory design to examine the hypothesized structural relationships among renewable energy literacy, personal energy values, energy attitudes, and energy-saving behavior. The conceptual framework is theory-informed rather than a complete operationalization of either VBN or TPB. Personal energy values represent the selected value-oriented component informed by VBN theory, whereas energy attitudes represent the selected attitudinal component informed by TPB. Normative, belief-based, perceived-control, and intention constructs specified in the complete theories are outside the scope of the present model. Energy-saving behavior is specified as a multidimensional second-order construct comprising three first-order subdimensions, namely curtailment behavior (ESB_CUR), efficiency behavior (ESB_EFF), and pro-social energy action (ESB_PRO), enabling simultaneous assessment of overall energy conservation behavior and its constituent behavioral domains.

The conceptual framework is provided as Figure 1 in the Figure legends section and as a separate figure file for submission.

**Figure 1.**
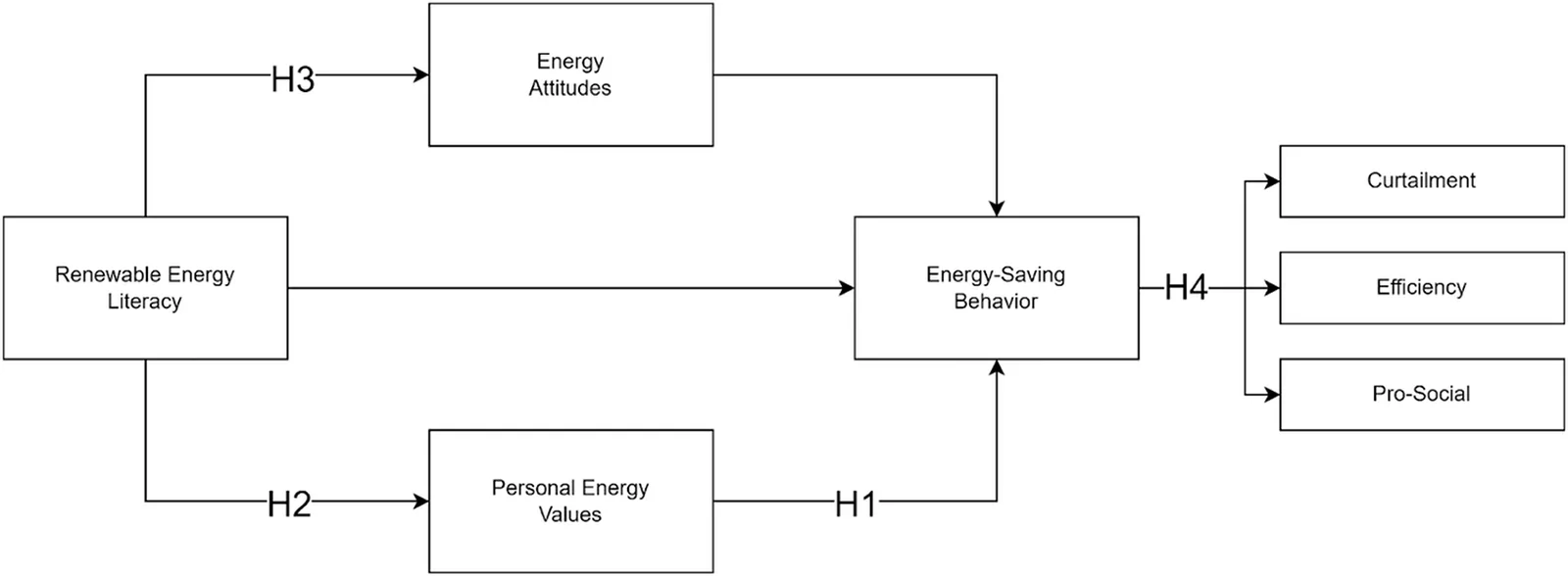
Theory-informed conceptual framework illustrating direct and indirect associations between renewable energy literacy and energy-saving behavior through personal energy values and energy attitudes. VBN and TPB are used selectively to inform the PEV and EA components, respectively; the framework does not represent the complete structure of either theory. REL → PEV → ESB (curtailment, efficiency, pro-social); REL → EA → ESB; REL → ESB (direct).

### Hypothesis development

**Renewable energy literacy and energy-saving behavior**

Energy literacy has evolved from a predominantly technical construct to a multidimensional conception integrating cognitive knowledge, affective engagement, and behavioral disposition ( DeWaters & Powers, 2013). In the present study, REL is operationalized as the extent to which individuals possess substantive understanding of renewable energy technologies, their environmental and economic implications, and the practical means through which energy-saving practices may be incorporated into daily and professional life. From an energy economics perspective, this operationalization frames REL as energy-related human capital: the informational foundation that equips individuals to recognize conservation opportunities and make informed decisions about energy use consistent with both private interests and broader social objectives ( Mireku et al., 2023).

Empirical evidence supports a positive association between energy literacy and energy-saving behavior across diverse national and institutional contexts ( Lee et al., 2015; Santillan & Cedano, 2023; Pothitou et al., 2015). Household-level studies confirm that energy-related knowledge is associated with stronger conservation practices, even when socioeconomic factors are controlled ( Brounen et al., 2013), while Indonesian evidence documents positive associations between energy knowledge and conservation behavior among citizens ( Hendinata, Ardiwinata, & Pratama, 2022) and educational attainment groups ( Endriana et al., 2025). However, observed REL–ESB effect sizes are frequently modest, reflecting the operation of mediating behavioral mechanisms.

**Personal energy values as a behavioral mediator**

VBN theory ( Stern, 2000) posits that pro-environmental behavior emerges through a motivational sequence in which biospheric and altruistic value orientations shape ecological beliefs and personal norms, which in turn motivate behavioral commitment. In the energy domain, these value orientations constitute internalized conservation preferences that shape individuals’ energy-related decision-making beyond the immediate calculus of personal economic benefit ( Kim & Kim, 2024). From a behavioral perspective, the cultivation of energy values through REL may help orient individual energy decisions more consistently with conservation objectives, providing a channel through which literacy contributes to demand-side behavioral change ( do Paço & Varejo, 2010).

Research indicates that REL may contribute to the development of energy-relevant value orientations ( Akitsu & Ishihara, 2018). Evidence for the mediating role of energy values in the literacy–behavior relationship has been provided by Appiah et al. (2023), who demonstrated that energy-related values partially transmitted the influence of energy literacy on energy-saving behavior. Pothitou et al. (2015) similarly identified environmental predisposition (a construct closely analogous to personal energy values) as a significant predictor of energy attitudes and behaviors. Cross-national evidence further confirms robust associations between environmental value orientations and energy-saving behavior ( Kim & Kim, 2024; Al Mamun et al., 2022).

**Energy attitudes as a behavioral mediator**

The Theory of Planned Behavior ( Ajzen, 1991) identifies attitudes as important proximal determinants of behavioral intention and action. In the energy conservation context, favorable energy attitudes reflect individuals’ positive evaluations of conservation practices, including their perceptions of the economic rationality, environmental significance, and personal importance of energy saving. Pothitou et al. (2015) demonstrated that energy attitudes were significantly correlated with energy-saving habits and behaviors, while do Paço and Varejo (2010) showed that attitudinal orientations toward the environment distinguished energy-saving consumers from non-savers. Across diverse institutional and cultural contexts, favorable attitudes toward energy conservation are consistently associated with stronger behavioral engagement ( Puiu et al., 2025; Xuan et al., 2023), and meta-analytic evidence confirms the central importance of attitudinal factors in explaining variations in conservation behavior ( Carrus et al., 2021).

In the proposed mediation model, energy attitudes function as an evaluative bridge between REL and ESB. Individuals with higher REL are expected to form more favorable evaluative orientations toward energy conservation, insofar as enhanced understanding of the environmental and economic consequences of energy use renders conservation both instrumentally rational and normatively desirable. Empirical support for this attitudinal mediation pathway has been provided by Appiah et al. (2023) and by household energy studies demonstrating that awareness-related factors influence conservation outcomes partly through attitudinal processes ( Gajdzik et al., 2024; Zhao, Song, & Wang, 2019).

### Multidimensional structure of energy-saving behavior

Energy-saving behavior encompasses multiple distinct behavioral domains that differ substantially in their motivational requirements, structural conditions, and determinant profiles ( Frederiks, Stenner, & Hobman, 2015). Following Abrahamse and Steg (2009), the present study distinguishes among: curtailment behaviors (habitual reductions in energy use achieved through everyday behavioral adjustments); efficiency behaviors (adoption of energy-efficient technologies requiring capital investment); and pro-social behaviors (advocacy and collective influence promoting conservation within social and professional networks).

These behavioral domains differ in their motivational requirements, structural conditions, and economic demands. Curtailment and pro-social behaviors generally involve behavioral adjustment and social engagement, whereas efficiency behaviors more frequently involve technological adoption and financial expenditure ( Steg & Vlek, 2009). These distinctions support treating ESB as a multidimensional construct rather than as a homogeneous behavioral measure. In the present study, the three domains are modeled as first-order components of a higher-order ESB construct; the analysis does not test formal differences in indirect effects among the subdimensions ( Duy et al., 2023; Frederiks et al., 2015). This distinction implies that behavioral literacy programs may be most effective in promoting curtailment and pro-social behaviors, while efficiency behaviors may require complementary economic instruments to overcome structural barriers ( Stancu et al., 2025).

### Setting, participants, and data collection

The study population comprised members of the Indonesia Fuel Cell and Hydrogen Energy Association (IFHE), a professional body representing individuals with direct engagement in renewable energy systems, hydrogen technologies, and related energy sector activities. This population was selected because it represents a professionally engaged renewable energy community with substantive exposure to energy-related issues. The study was designed to examine the proposed relationships within this professional context rather than to estimate patterns representative of the Indonesian general population. Given the specialized composition of the sample, the distribution of REL may be more restricted than in a population-based sample, and the possibility of ceiling effects cannot be excluded. Accordingly, the findings should be interpreted as evidence pertaining to IFHE members and similar professionally engaged energy communities. From an energy economics perspective, energy sector professionals remain strategically relevant because their conservation practices, value orientations, and energy attitudes may shape organizational energy management and the diffusion of conservation practices within professional networks.

Purposive sampling was employed to ensure the requisite domain knowledge among respondents. The study commenced and data collection began on 29 April 2026, after ethical approval had been obtained. Data were collected through a combination of online and in-person survey administration to maximize coverage within the IFHE membership. Before participating, all respondents received written information explaining the research purpose, the voluntary nature of participation, response anonymity, data confidentiality, and their right to decline or withdraw from the study. Written informed consent was obtained electronically from all participants by requiring them to select the consent button before accessing and completing the questionnaire. This consent procedure was included in and approved as part of the study’s ethics protocol. Following data screening for completeness and response quality, a final usable sample of 195 valid responses was obtained.

### Variables and measurement

All constructs were operationalized using a structured questionnaire with items rated on a four-point forced-choice Likert scale (1 = strongly disagree; 4 = strongly agree). The elimination of a neutral midpoint was designed to reduce acquiescence bias and encourage directional response. The complete wording of all questionnaire items, item codes, adaptation status, and source references is provided in Extended Data Table S1 to make the operational meaning of each latent construct transparent.

Renewable Energy Literacy (REL) was measured using four items adapted from DeWaters and Powers (2013), reflecting respondents’ knowledge, understanding, and awareness of renewable energy systems and their implications for energy use and conservation, capturing cognitive dimensions including knowledge of energy sources, energy system functioning, and sustainability-related energy implications.

Personal Energy Values (PEV) were measured using four items capturing biospheric and altruistic value orientations relevant to energy use and environmental sustainability, grounded in VBN theory ( Stern, 2000), consistent with established operationalizations in the energy conservation literature ( Al Mamun et al., 2022; Appiah et al., 2023).

Energy Attitudes (EA) were assessed using three items reflecting respondents’ cognitive and affective evaluations of energy conservation practices, adapted from established TPB-based attitudinal instruments ( Ajzen, 1991; Puiu et al., 2025).

Energy-Saving Behavior (ESB) was operationalized as a multidimensional second-order construct comprising nine items across three first-order subdimensions: curtailment behavior (ESB_CUR; three items), efficiency behavior (ESB_EFF; three items), and pro-social energy action (ESB_PRO; three items), grounded in the taxonomy of Abrahamse and Steg (2009). The measurement items, their sources, and their analytical status are presented in Table 1. The questionnaire underwent pilot testing prior to full deployment to verify semantic clarity, cultural appropriateness, and content validity.

**Table 1.** Measurement items, sources, and analytical status.

Construct	Code	Questionnaire item	Source/adaptation	Analytical status
Renewable Energy Literacy (REL)	REL1	I am able to distinguish credible energy information from biased information.	DeWaters & Powers (2013)	Retained
Renewable Energy Literacy (REL)	REL2	I am able to compare renewable energy and fossil energy in terms of cost and environmental impact.	U.S. DoE (2012); Lowan-Trudeau & Fowler (2021)	Retained
Renewable Energy Literacy (REL)	REL3	I am able to estimate the long-term cost of using electrical appliances before purchasing them.	DeWaters & Powers (2013)	Retained
Renewable Energy Literacy (REL)	REL4	I am willing to change my views about energy based on new and trustworthy evidence.	Cotton et al. (2015)	Retained
Energy Attitudes (EA)	ATT1	Reducing energy consumption is an important priority for me.	DeWaters & Powers (2013)	Retained
Energy Attitudes (EA)	—	I feel personally responsible for the impacts of energy use.	Stern (2000)	Administered but excluded from the final measurement model
Energy Attitudes (EA)	ATT2	I believe that my individual actions can make a difference when performed consistently.	DeWaters & Powers (2013)	Retained
Energy Attitudes (EA)	ATT3	I believe that collective community action can accelerate the energy transition.	Wüstenhagen et al. (2007)	Retained
Personal Energy Values (PEV)	VAL1	I choose products that do not harm the environment.	Stern (2000)	Retained
Personal Energy Values (PEV)	VAL2	I consider environmental impacts when making consumption decisions.	Stern (2000)	Retained
Personal Energy Values (PEV)	VAL3	I am concerned about the waste of the planet’s resources.	Stern (2000)	Retained
Personal Energy Values (PEV)	VAL4	I see myself as responsible for the environment.	Stern (2000)	Retained
ESB – Curtailment (ESB_CUR)	CUR1	I switch off electronic devices when they are not in use.	Gardner & Stern (2002); Steg & Vlek (2009)	Retained
ESB – Curtailment (ESB_CUR)	CUR2	I adjust the temperature or duration of air-conditioner use to reduce electricity consumption.	Steg & Vlek (2009)	Retained
ESB – Curtailment (ESB_CUR)	CUR3	I routinely unplug chargers or electronic devices from electrical outlets when they are not in use.	Gardner & Stern (2002)	Retained
ESB – Efficiency (ESB_EFF)	EFF1	I replace old appliances with more energy-efficient ones whenever possible.	Gardner & Stern (2002); Lange Salvia et al. (2020)	Retained
ESB – Efficiency (ESB_EFF)	EFF2	When purchasing appliances, I check and compare their energy-consumption labels.	DeWaters & Powers (2013)	Retained
ESB – Efficiency (ESB_EFF)	EFF3	I perform regular maintenance so that appliances such as air conditioners and refrigerators operate more efficiently.	Steg & Vlek (2009)	Retained
ESB – Pro-Social (ESB_PRO)	PRO1	I encourage other people to save energy.	Cotton et al. (2015)	Retained
ESB – Pro-Social (ESB_PRO)	PRO2	I share the energy-saving practices I use with family members or friends.	Abrahamse & Matthies (2019)	Retained
ESB – Pro-Social (ESB_PRO)	PRO3	I support or participate in energy-saving campaign initiatives.	Cotton et al. (2015)	Retained

### Statistical analysis

Data were analyzed using Partial Least Squares Structural Equation Modeling (PLS-SEM) implemented in SmartPLS 4. PLS-SEM was selected because the proposed model combines multiple indirect relationships with a hierarchical multidimensional ESB construct and permits simultaneous estimation of relationships among latent constructs. The analytical objective was primarily explanatory rather than out-of-sample predictive; therefore, predictive performance and distributional non-normality were not used as primary justifications for selecting PLS-SEM ( Hair, Hult, Ringle, & Sarstedt, 2017). The analysis proceeded through a two-stage sequential approach comprising measurement model assessment followed by structural model evaluation.

The analysis followed a disjoint two-stage approach for the higher-order ESB construct. In the first stage, REL, PEV, EA, ESB_CUR, ESB_EFF, and ESB_PRO were estimated from their respective reflective indicators. Construct reliability was evaluated using Cronbach’s alpha (α ≥ .70) and composite reliability (CR ≥ .70). Convergent validity was assessed through standardized indicator loadings (λ ≥ .70) and Average Variance Extracted (AVE ≥ .50), while discriminant validity was examined using the Fornell–Larcker criterion and the heterotrait–monotrait ratio (HTMT < .90) ( Hair et al., 2017).

ESB was specified as a reflective–reflective hierarchical component model. In the second stage of the disjoint two-stage procedure, the latent variable scores of ESB_CUR, ESB_EFF, and ESB_PRO obtained from the first stage were used as indicators of the higher-order ESB construct. The original lower-order item indicators were therefore not repeated at the higher-order stage. The structural model was subsequently evaluated through path coefficients (β), coefficients of determination (R ^2^), and effect sizes (f ^2^). Statistical significance of direct and indirect associations was assessed via bootstrapping with 5,000 resamples. Mediation was summarized using the Variance Accounted For (VAF) index; values between 20% and 80% were interpreted as partial mediation, while values exceeding 80% were interpreted as full mediation ( Hair et al., 2017).

### Preregistered data analysis

This study was not preregistered. The analysis plan was developed before the final statistical analysis was conducted, but it was not registered in an independent public registry.

## Results

### Respondent profile

Table 2 presents the demographic profile of respondents. The sample is predominantly male (n = 168, 86.2%), concentrated in the 25–34 age cohort (n = 91, 46.7%), and reflects the professional renewable energy community in Indonesia. Most respondents hold undergraduate (55.9%) or postgraduate (25.1%) qualifications. Household income is most frequently concentrated in the 5–10 million IDR range (50.8%), with 21.0% in the 10–20 million IDR range, consistent with the middle-income professional demographic of Indonesia’s urban energy sector. This income distribution is economically relevant: the predominance of middle-income respondents reflects economic constraints that may limit capital-intensive energy efficiency investment even among professionally energy-literate individuals.

**Table 2.** Demographic profile of respondents (N = 195).

Variable	Category	n	%
Gender	Male	168	86.2
Gender	Female	27	13.8
Age	25–34 years	91	46.7
Age	35–44 years	47	24.1
Age	45–54 years	30	15.4
Age	>55 years	13	6.7
Marital Status	Married	138	70.8
Marital Status	Unmarried	57	29.2
Education	High school	37	19.0
Education	Undergraduate	109	55.9
Education	Postgraduate	49	25.1
Household Income	< 5 million IDR	36	18.5
Household Income	5–10 million IDR	99	50.8
Household Income	10–20 million IDR	41	21.0
Household Income	> 20 million IDR	8	4.1
Household Income	Prefer not to disclose	11	5.6

### Measurement model assessment

Table 3 presents discriminant validity results using the Fornell–Larcker criterion. The square root of the AVE for each construct exceeded its correlations with the other constructs. Discriminant validity was additionally assessed using the heterotrait–monotrait ratio (HTMT), with all construct pairs below the .90 threshold. The complete HTMT matrix is reported in Extended Data Table S2. Together, these results provide evidence that REL, PEV, EA, and the ESB dimensions are empirically distinguishable within the present sample.

**Table 3.** Measurement model results.

Construct	Indicator	Loading	Cronbach’s α	Comp. Reliability	AVE
Energy Attitudes (EA)	ATT1	0.802	0.628	0.647	0.577
Energy Attitudes (EA)	ATT2	0.641			
Energy Attitudes (EA)	ATT3	0.823			
ESB — Curtailment (ESB_CUR)	CUR1	0.760	0.602	0.790	0.556
ESB — Curtailment (ESB_CUR)	CUR2	0.719			
ESB — Curtailment (ESB_CUR)	CUR3	0.758			
ESB — Efficiency (ESB_EFF)	EFF1	0.876	0.839	0.903	0.757
ESB — Efficiency (ESB_EFF)	EFF2	0.831			
ESB — Efficiency (ESB_EFF)	EFF3	0.901			
ESB — Pro-Social (ESB_PRO)	PRO1	0.851	0.704	0.835	0.629
ESB — Pro-Social (ESB_PRO)	PRO2	0.786			
ESB — Pro-Social (ESB_PRO)	PRO3	0.740			
Renewable Energy Literacy (REL)	REL1	0.791	0.740	0.837	0.563
Renewable Energy Literacy (REL)	REL2	0.694			
Renewable Energy Literacy (REL)	REL3	0.693			
Renewable Energy Literacy (REL)	REL4	0.815			
Personal Energy Values (PEV)	VAL1	0.834	0.785	0.862	0.611
Personal Energy Values (PEV)	VAL2	0.780			
Personal Energy Values (PEV)	VAL3	0.694			
Personal Energy Values (PEV)	VAL4	0.811			

*Note:* Loadings below.70 were retained based on substantive contribution to construct coverage. Cronbach’s alpha for ESB_CUR (α = .602) and EA (α = .628) fall below the conventional.70 threshold; however, composite reliability and AVE values for all constructs meet established criteria ( Hair et al., 2017), justifying retention of these measurement specifications. Readers should note that the composite reliability of EA (CR = 0.647) is also marginally below the conventional threshold; the substantive interpretation of EA-mediated findings should be considered in light of this measurement limitation.

Table 4 presents discriminant validity results using the Fornell–Larcker criterion. The square root of the AVE for each construct (diagonal values in bold) exceeds its correlations with all other constructs, confirming the empirical distinctiveness of each measurement instrument. Heterotrait-monotrait ratio (HTMT) values were below the .90 threshold for all construct pairs, providing additional confirmation of satisfactory discriminant validity.

**Table 4.** Discriminant validity — Fornell–Larcker criterion.

Construct	EA	ESB	ESB_CUR	ESB_EFF	ESB_PRO	REL	PEV
EA	0.759						
ESB	0.624	0.621					
ESB_CUR	0.639	0.806	0.746				
ESB_EFF	0.345	0.682	0.289	0.870			
ESB_PRO	0.473	0.845	0.594	0.342	0.793		
REL	0.574	0.599	0.579	0.230	0.573	0.750	
PEV	0.517	0.677	0.674	0.352	0.549	0.586	0.781

*Note:* Diagonal values (bold) represent the square root of AVE; off-diagonal values are inter-construct correlations. EA = Energy Attitudes; ESB = Energy-Saving Behavior; REL = Renewable Energy Literacy; PEV = Personal Energy Values.

The measurement model diagram is provided as Figure 2 in the Figure legends section and as a separate figure file for submission.

**Figure 2.**
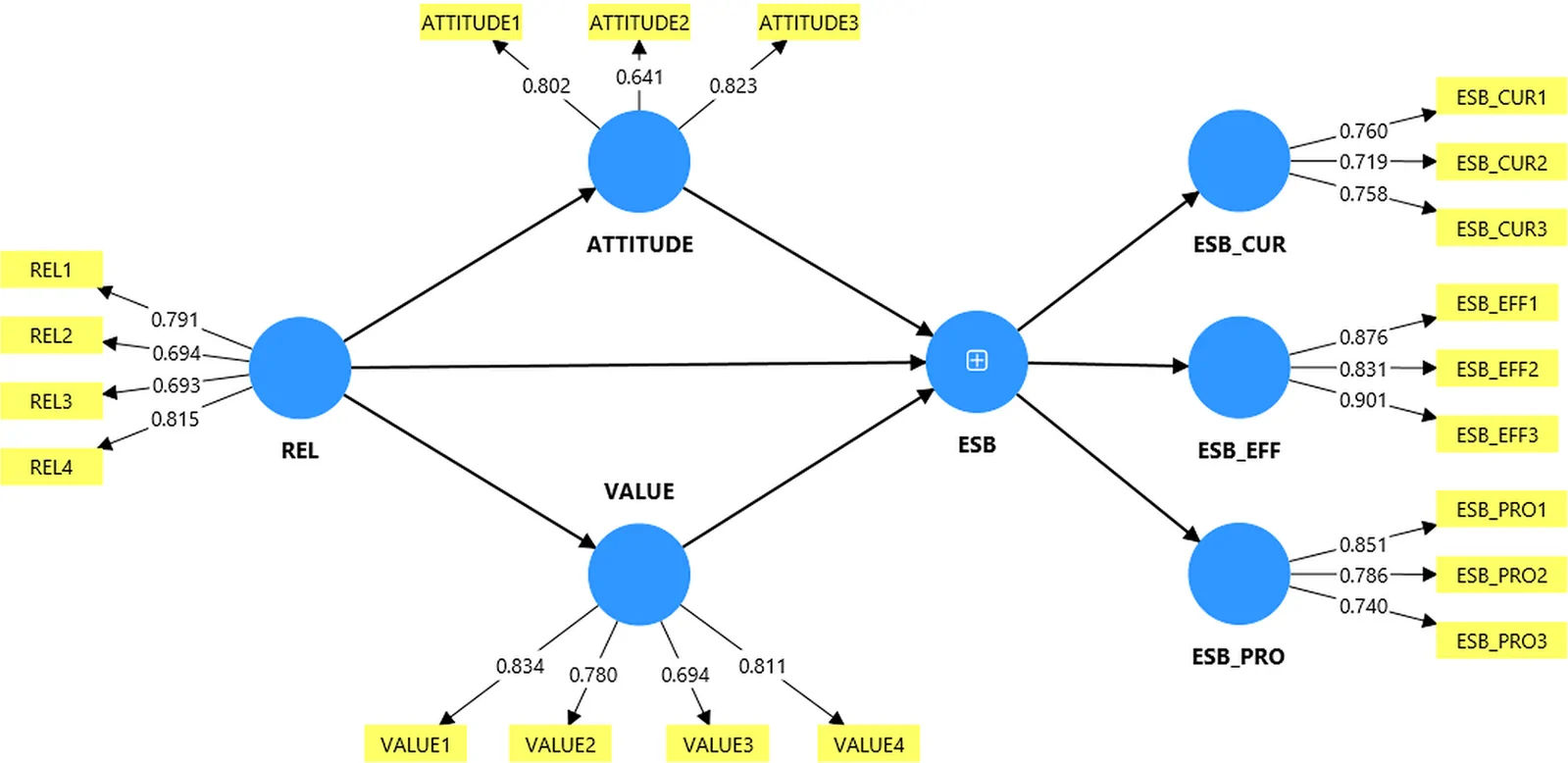
Measurement model diagram (outer loadings).

### Structural model assessment

Table 5 presents the structural model results. REL was positively associated with both PEV (β = 0.586, t = 10.238, p < .001, f ^2^ = 0.522) and EA (β = 0.574, t = 9.915, p < .001, f ^2^ = 0.491). PEV and EA were also positively associated with ESB, with PEV showing β = 0.411 (t = 5.685, p < .001, f ^2^ = 0.244) and EA showing β = 0.307 (t = 4.306, p < .001, f ^2^ = 0.139). The direct association between REL and ESB remained statistically significant but modest (β = 0.182, t = 2.273, p = .023, f ^2^ = 0.044). The model accounted for 57.9% of the variance in ESB (R ^2^ = 0.579).

**Table 5.** Structural model results.

Path	β	t-value	p-value	R ^2^	f ^2^	Effect Size
REL → EA	0.574	9.915	<.001	0.329	0.491	Medium
REL → PEV	0.586	10.238	<.001	0.343	0.522	Large
REL → ESB (direct)	0.182	2.273	.023	0.579	0.044	Small
EA → ESB	0.307	4.306	<.001	—	0.139	Small
PEV → ESB	0.411	5.685	<.001	—	0.244	Medium
ESB → ESB_CUR	0.806	33.531	<.001	0.650	1.859	Large
ESB → ESB_EFF	0.682	7.894	<.001	0.465	0.870	Large
ESB → ESB_PRO	0.845	33.803	<.001	0.714	2.497	Large

*Note:* REL = Renewable Energy Literacy; EA = Energy Attitudes; PEV = Personal Energy Values; ESB = Energy-Saving Behavior; β = standardized path coefficient; β ^2^ = effect size; R ^2^ = coefficient of determination.

The structural model is provided as Figure 3 in the Figure legends section and as a separate figure file for submission.

**Figure 3.**
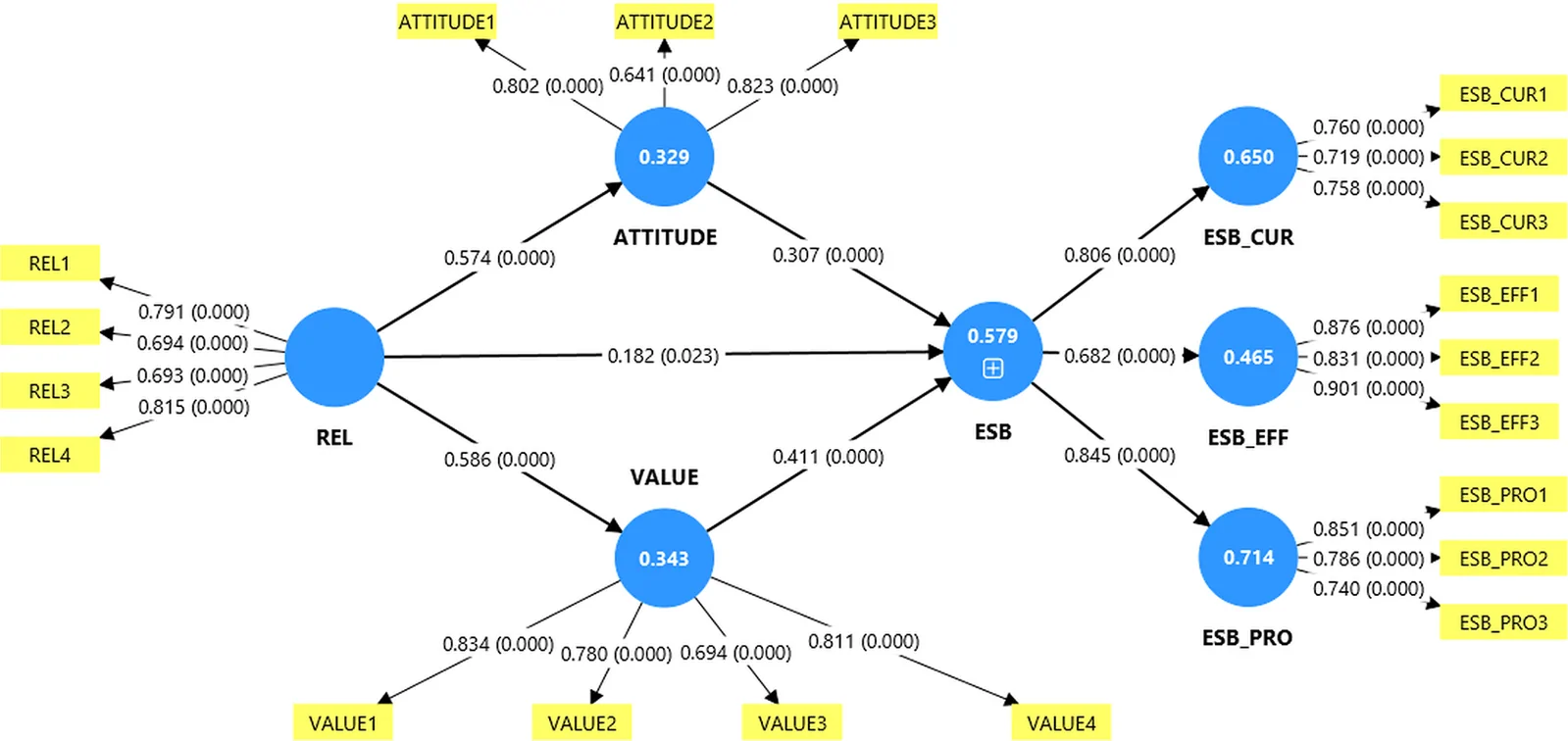
Structural model.

### Hypothesis testing and mediation analysis

Table 6 presents the mediation analysis results. The total association between REL and ESB was statistically significant (β = 0.599, t = 11.371, p < .001). The direct association was β = 0.182, while significant indirect associations were observed through PEV (β = 0.241, p < .001) and EA (β = 0.176, p < .001). The combined indirect component accounted for a substantial proportion of the modeled REL–ESB association, consistent with partial mediation within the specified structural model. H1, H2, and H3 were supported. Because the higher-order ESB specification was used to represent the three behavioral dimensions jointly, no inferential claim is made regarding statistically significant differences in mediation across curtailment, efficiency, and pro-social subdimensions.

**Table 6.** Mediation analysis results.

Path	Direct β	Indirect β	Total β	t-value	p-value	VAF (%)	Mediation
REL → ESB	0.182	0.417	0.599	11.371	<.001	69.6%	Partial
REL → PEV → ESB	—	0.241	—	5.008	<.001	57%	Partial
REL → EA → ESB	—	0.176	—	4.681	<.001	49%	Partial
PEV → ESB	0.411	—	0.411	5.685	<.001	—	Direct
EA → ESB	0.307	—	0.307	4.306	<.001	—	Direct

*Note:* VAF = Variance Accounted For. REL = Renewable Energy Literacy; PEV = Personal Energy Values; EA = Energy Attitudes; ESB = Energy-Saving Behavior.

## Discussion

Before interpreting the structural relationships, the characteristics of the study population should be considered. IFHE members represent a specialized professional community with direct exposure to renewable energy issues; therefore, their literacy, values, attitudes, and energy-saving behavior may differ from those of Indonesian households or the general public. The estimated relationships should consequently be interpreted within this professional context. The specialized sample may also restrict variation in REL and may produce ceiling effects that were not directly assessed in the present study. Replication with broader and more heterogeneous population samples is needed before extending the findings to the Indonesian public.

### Renewable energy literacy as a demand-side behavioral determinant

The statistically significant but modest direct association between REL and ESB (β = 0.182, p = .023, f ^2^ = 0.044) supports H1 and indicates that higher renewable energy literacy is associated with stronger energy-saving behavior within the IFHE sample. From a behavioral energy economics perspective, this pattern is consistent with viewing REL as energy-related human capital: an informational resource that may accompany more informed energy decisions while not by itself establishing behavioral change ( Mireku et al., 2023). Just as financial literacy creates the informational conditions for sound financial decisions without guaranteeing optimal behavioral outcomes, energy literacy equips individuals with the cognitive foundation for conservation while requiring complementary motivational and evaluative processes to generate actual behavioral change.

This interpretation is consistent with substantial prior evidence on the knowledge–behavior gap. do Paço and Varejo (2010) demonstrated that even among environmentally concerned consumers, energy knowledge did not reliably distinguish energy savers from non-savers; rather, it was the economic cost of electricity that most powerfully differentiated these behavioral groups, suggesting that knowledge becomes behaviorally consequential primarily when accompanied by economically relevant evaluations and motivations. Pothitou et al. (2015) similarly found that the path from knowledge to actual conservation behavior was considerably weaker than the attitude–behavior pathway, a pattern directly mirrored in the present study’s finding that REL’s indirect effects through PEV and EA (β = 0.417) substantially exceed the direct effect (β = 0.182).

The relatively small direct effect size (f ^2^ = 0.044) has an implication for Indonesia’s demand-side energy management agenda: energy literacy campaigns that deliver knowledge without simultaneously cultivating conservation values and favorable attitudes are unlikely to generate the sustained behavioral change needed to contribute meaningfully to household energy demand reduction. The significant residual direct effect (β = 0.182, p = .023) nevertheless indicates that REL makes a behavioral contribution beyond what is captured by the proposed mediators, reflecting proximal cognitive processes (including enhanced awareness of energy-saving opportunities and increased behavioral self-efficacy) that operate independently of value and attitudinal channels.

### Personal energy values in the REL–ESB association

The significant indirect association between REL and ESB through personal energy values (β = 0.241, VAF = 57%) supports H2 within the specified model and is consistent with a value-oriented relationship linking energy literacy and conservation behavior. This pattern extends the prior evidence of Appiah et al. (2023) and is consistent with Pothitou et al.’s (2015) finding that environmental predisposition was associated with both environmental knowledge and conservation behavior. Cross-national evidence also documents positive associations between environmental value orientations and energy-saving behavior across diverse institutional settings ( Kim & Kim, 2024; Al Mamun et al., 2022).

The VAF estimate indicates that PEV accounts for a substantial share of the modeled indirect association between REL and ESB. However, this result should not be interpreted as evidence that REL temporally changes personal energy values or that such value change subsequently causes energy-saving behavior because all constructs were measured at the same point in time. Rather, the findings show that REL, PEV, and ESB are related in a pattern consistent with the proposed mediation structure.

An economically significant implication concerns the potential durability of literacy-driven conservation outcomes. Prior research suggests that values-based motivations may be relatively stable across varying economic conditions ( do Paço & Varejo, 2010). If so, individuals with stronger conservation values cultivated through REL may maintain conservation behavior more consistently than those motivated primarily by economic factors. The present cross-sectional data do not directly test this longitudinal inference, but the findings are consistent with this interpretation.

### Energy attitudes as an evaluative pathway for conservation behavior

The significant indirect association between REL and ESB through energy attitudes (β = 0.176, VAF = 49%) supports H3 within the specified model and is consistent with an evaluative relationship linking renewable energy literacy and energy-saving behavior. This pattern is consistent with TPB’s identification of attitudes as an important correlate of behavioral intention and action ( Ajzen, 1991) and with prior energy-conservation research documenting associations between favorable energy attitudes and stronger behavioral engagement ( Pothitou et al., 2015; Puiu et al., 2025; Xuan et al., 2023).

The observed indirect association should nevertheless be interpreted within the limitations of the cross-sectional design. The present data do not establish that higher REL causes more favorable energy attitudes or that attitudinal change subsequently produces energy-saving behavior. Instead, the findings indicate that REL, EA, and ESB are statistically related in a pattern consistent with the proposed mediation model.

### Policy implications for professional energy communities

The findings have their most direct practical relevance for professional renewable energy communities rather than for the Indonesian general public. Within organizations and professional associations whose members already have substantive exposure to energy issues, literacy-oriented initiatives may be strengthened by incorporating value-oriented and attitudinal components alongside technical information. Associations such as IFHE and energy-sector organizations may use these findings to inform professional development, internal energy-management communication, and peer-based conservation initiatives. The significant indirect associations through PEV and EA suggest that professional energy programs may consider not only knowledge acquisition but also the value orientations and evaluative attitudes accompanying conservation behavior. However, the present study does not establish that interventions targeting these factors will cause behavioral change. The findings instead identify statistically supported relationships within a cross-sectional professional sample that may inform the design of future intervention studies. Because the sample is not population-based, these implications should not be extended directly to Indonesian households or the general public. Broader policy recommendations require replication in samples with greater variation in energy literacy, socioeconomic conditions, and access to energy-saving technologies ( IEA, 2023; Yildiz, 2026).

Second, the dual-pathway finding, in which PEV (VAF = 57%) and EA (VAF = 49%) jointly account for approximately 70% of the total REL–ESB relationship, implies that demand-side programs should target both behavioral pathways simultaneously. Values-based interventions delivered through sustained professional community engagement can cultivate stable motivational orientations generating durable conservation behavior. Attitude-based interventions, including economic framing of conservation benefits and social comparison programs, can shift evaluative orientations more rapidly and may produce shorter-term behavioral returns.

Third, the comparatively weaker response observed for efficiency-related behaviors suggests the need for a complementary economic policy architecture alongside behavioral programs. For efficiency-related behaviors, the findings are consistent with the possibility that financial and structural barriers limit the effectiveness of literacy-driven interventions for efficiency-related behaviors. Economic instruments including appliance efficiency subsidies, tax credits for energy-efficient investments, low-interest green financing for energy-sector professionals, and minimum efficiency standards are essential complements in the Indonesian context ( Duy et al., 2023; IEA, 2023; Hardi et al., 2024).

## Conclusions

This study examined the direct and indirect associations between renewable energy literacy and multidimensional energy-saving behavior among members of the Indonesia Fuel Cell and Hydrogen Energy Association using a theory-informed framework drawing selectively on VBN theory and TPB. REL was positively associated with ESB directly and indirectly through personal energy values and energy attitudes within the specified cross-sectional model. These results indicate that value orientations and energy attitudes are important correlates of the REL–ESB relationship among professionally engaged energy-sector participants. The study contributes to behavioral energy research by representing energy-saving behavior as a multidimensional higher-order construct encompassing curtailment, efficiency, and pro-social behavior. However, the present analysis does not establish statistically significant differences in mediated associations across these subdimensions, and no such comparative claim is made. The results therefore support interpretation at the level of the higher-order ESB construct rather than differential mediation across its components.

From a practical perspective, the findings may inform future professional energy-literacy initiatives that consider knowledge together with value and attitudinal dimensions. These implications are most directly applicable to professional renewable energy communities similar to IFHE. They should not be generalized directly to the Indonesian public or interpreted as evidence that literacy-based interventions will causally change energy-saving behavior.

Several limitations should be acknowledged. First, the cross-sectional design limits temporal and causal inference; the indirect effects identified in the model should therefore be interpreted as statistical indirect associations rather than evidence of causal mechanisms. Second, the professional composition of the IFHE sample constrains generalizability to the Indonesian general public and may restrict variation in renewable energy literacy, including the possibility of ceiling effects that were not directly assessed. Third, the use of self-reported measures introduces the possibility of response bias. Future research should employ broader population-based samples, longitudinal designs, objective energy-consumption indicators, and economic variables such as energy expenditure, price sensitivity, and income to further advance understanding of literacy-related conservation behavior in energy transition contexts.

## Ethical considerations

This study was conducted in accordance with ethical principles for research involving human participants. The study protocol was approved by Universitas Sebelas Maret, approval number 11729/UN27.02/PT.01.04/2026, on 27 April 2026. The study commenced on 29 April 2026, after ethical approval had been obtained. Before participating, all respondents received written information explaining the research purpose, the voluntary nature of participation, response anonymity, data confidentiality, and their right to decline or withdraw from the study. Written informed consent was obtained electronically from all participants by requiring them to select the consent button before accessing and completing the questionnaire. This consent procedure was included in and approved as part of the study’s ethics protocol. No personal identifiers were collected or retained in the analytical dataset.

## Use of artificial intelligence (AI)

Generative AI tools were used to assist with language editing, drafting, and structural refinement of the manuscript. No AI tools were used for data collection, statistical analysis, interpretation of results, or generation of research findings. All intellectual content, analysis, and conclusions represent the authors’ own work and responsibility.

## Data Availability

The individual-level survey data underlying the results of this study are not publicly available under an open licence because respondents consented to participation in this study and to restricted academic or verification access only; the consent process did not include unrestricted public deposition or public reuse of participant-level survey data. Public release could create a risk of deductive disclosure because the dataset was obtained from a defined professional community, the Indonesia Fuel Cell and Hydrogen Energy Association (IFHE), and includes demographic and professional characteristics that may be identifiable in small subgroups. The restriction therefore applies to public deposition and unrestricted reuse of participant-level data; no direct personal identifiers were collected or retained in the analytical dataset. The Universitas Sebelas Maret ethics approval did not authorize unrestricted public sharing of participant-level survey data. To support transparency and verification, de-identified intermediary materials that do not compromise participant anonymity may be made available on reasonable request, including aggregate descriptive outputs, the anonymized variable dictionary/codebook, the questionnaire and measurement items, and the SmartPLS output used to support the reported tables. A de-identified participant-level dataset may be considered for access only where the request is for reasonable academic or verification purposes and where disclosure does not compromise participant anonymity. The extended materials supporting this study, including the survey questionnaire, measurement items, data dictionary/codebook, SmartPLS output, and reporting checklist, are not deposited publicly because these materials are linked to the restricted survey dataset and study documentation. Extended materials that can be shared without compromising anonymity may be requested from the corresponding author for reasonable academic and verification purposes, subject to the confidentiality and data-use conditions described below. Requests for underlying data or extended materials should be sent to the corresponding author, Dewi Kusuma Wardani ( dewikusuma@staff.uns.ac.id). Requests should include the requester’s name, institutional affiliation, research purpose, proposed analysis plan, exact data or materials requested, ethics approval or exemption where applicable, and a statement confirming that the requester will not attempt to re-identify participants, redistribute the data or materials, or use them for purposes other than those approved by the authors. Access will be considered by the corresponding author and research team and may be granted subject to ethical, privacy, confidentiality, and institutional requirements, including institutional or ethics approval where required and a signed data-use or confidentiality agreement. Data analysis was conducted using SmartPLS 4. No custom software or original programming code was developed for this study. The analysis used standard SmartPLS procedures for measurement model assessment, structural model assessment, bootstrapping, and mediation analysis. SmartPLS project files and statistical output files may be requested from the corresponding author for reasonable academic and verification purposes, subject to the confidentiality and data-use conditions described in the Data availability section. This cross-sectional survey study was reported in accordance with the STROBE reporting guideline for observational studies. The completed STROBE checklist may be requested from the corresponding author for reasonable academic and verification purposes, subject to the conditions described in the Data availability section.
