# Supplementary material for: Renewable Energy Literacy and Multidimensional Energy-Saving Behavior in Indonesia: The Roles of Personal Energy Values and Energy Attitudes
Source: F1000Res. 2026 Nov 9;15:1250. Originally published 2026 Jul 29. [Version 2] doi: 10.12688/f1000research.185821.2 (PMC13560042; doi:10.12688/f1000research.185821.2)
Supplement: Peer review history

# Reviewer report: Reviewer response for version 1

**DOI:** 10.5256/f1000research.205102.r507437

**Published:** 2026-08-21

**Authors:** Keiichi Ishihara, Yutaka Akitsu

**Affiliations:** 1 Kyoto University, Sakyo, Japan; 2 Research Institute of Energy Literacy, Tokyo, Japan

This paper reports on statistical considerations regarding the relationship between renewable literacy and energy-saving behavior, using structural equation modeling. In particular, the authors distinguish energy-saving behaviors into curtailment, pro-social behaviors, and efficiency behaviors. There are some drawbacks in this manuscript. The reviewers suggest some points to improve the paper.

 1. The survey participants

 The survey participants are limited to members of the Indonesia Fuel Cell and Hydrogen Energy Association (IEHE). Although it states "while mitigating ceiling effects," it seems likely that ceiling effects do exist. Therefore, the results are quite limited, making generalization difficult, and policy recommendations targeting the general public are certainly not warranted.

 2. The model provided by the authors (Figure 1)

 The authors explain the model in terms of the two well-known models, VBN and TPB. However, this model is quite different from these models. These two models emphasize the importance of norms. However, the authors ignore the concepts of these models. The reviewer requests that the authors explain the relationship between the model (Fig. 1) and the referenced models (VBN and TPB).

 3. The meaning of variables

 Each latent variable has an important meaning and consists of several observed variables. Since the paper does not show the questions corresponding to the observed variables, the true meaning of the latent variables remains unclear. We strongly recommend that the complete questionnaire items and their sources be provided. The manuscript also states that discriminant validity was assessed using the HTMT and cross-loadings. Providing these results would help readers evaluate whether conceptually related constructs such as REL, PEV, and EA are empirically distinguishable.

 4. The analysis of SEM

 For example, non-normality is cited as a reason for selecting PLS-SEM, although evidence demonstrating non-normality of the present data is not reported. The higher-order ESB specification should also be described more explicitly, including the reflective/formative specification of the higher-order construct and the implementation of the disjoint two-stage approach. If predictive performance is part of the rationale for selecting PLS-SEM, the authors should consider reporting an appropriate out-of-sample predictive assessment (e.g., PLSpredict), including prediction errors such as RMSE/MAE and comparison with a linear-model benchmark. This would provide study-specific evidence for the predictive rationale underlying the choice of PLS-SEM.

 5. The meaning of the model

 The authors stated that mediated effects are stronger for curtailment and pro-social behaviors than for efficiency behavior. However, the differences are narrow. Appropriate bootstrap contrasts, such as the differences between the pro-social and efficiency indirect effects and between the curtailment and efficiency indirect effects, are needed before H4 can be considered statistically supported. The calculation of these sub dimensional indirect effects should also be explained clearly, particularly with respect to the second-order ESB specification. Because this study is cross-sectional, the significant indirect effects through PEV and EA should be interpreted as indirect associations within the specified model rather than as evidence of a temporal or causal mechanism. The conclusion should reflect the limitation of this study.

Is the work clearly and accurately presented and does it cite the current literature?

Partly

If applicable, is the statistical analysis and its interpretation appropriate?

Partly

Are all the source data underlying the results available to ensure full reproducibility?

No

Is the study design appropriate and is the work technically sound?

Partly

Are the conclusions drawn adequately supported by the results?

Partly

Are sufficient details of methods and analysis provided to allow replication by others?

Partly

Reviewer Expertise:

Energy system analysis, Energy policy, Energy education, Energy literacy

We confirm that we have read this submission and believe that we have an appropriate level of expertise to confirm that it is of an acceptable scientific standard, however we have significant reservations, as outlined above.

# Reviewer report: Reviewer response for version 1

**DOI:** 10.5256/f1000research.205102.r507433

**Published:** 2026-08-07

**Authors:** Dedi Rohendi

**Affiliations:** 1 Universitas Sriwijaya, Indralaya, Indonesia

This manuscript investigates the relationships between renewable energy literacy (REL), personal energy values (PEV), energy attitudes (EA), and multidimensional energy-saving behavior (ESB) among members of the Indonesia Fuel Cell and Hydrogen Energy Association (IFHE). Using a Partial Least Squares Structural Equation Modeling (PLS-SEM) approach, the authors demonstrate that personal energy values and energy attitudes partially mediate the influence of renewable energy literacy on energy-saving behavior.

 The manuscript addresses an important and timely topic considering Indonesia's ongoing energy transition and increasing attention to demand-side energy management. The theoretical integration of the Value-Belief-Norm (VBN) Theory and the Theory of Planned Behavior (TPB) is appropriate, and the manuscript is generally well organized and clearly written.

 Overall, this is a valuable contribution to the literature. Several issues should be addressed or added before indexing:

1. The study exclusively surveyed members of the Indonesia Fuel Cell and Hydrogen Energy Association (IFHE), who already possess relatively high knowledge regarding renewable energy. The authors acknowledge this limitation in the conclusion; however, it should also **be emphasized earlier in the Discussion section.**
2. The literature review is comprehensive and up to date. However, several paragraphs are relatively long. **Shortening some sections would improve readability.**
3. Figures 2 and 3 appear to have been prepared directly from SmartPLS. Please improve the graphical quality and add the appropriate captions for these figures.

Is the work clearly and accurately presented and does it cite the current literature?

Yes

If applicable, is the statistical analysis and its interpretation appropriate?

I cannot comment. A qualified statistician is required.

Are all the source data underlying the results available to ensure full reproducibility?

Yes

Is the study design appropriate and is the work technically sound?

Yes

Are the conclusions drawn adequately supported by the results?

Yes

Are sufficient details of methods and analysis provided to allow replication by others?

Yes

Reviewer Expertise:

Fuel cell and hydrogen technology, new and renewable energy policy

I confirm that I have read this submission and believe that I have an appropriate level of expertise to confirm that it is of an acceptable scientific standard.

# Reviewer report: Reviewer response for version 1

**DOI:** 10.5256/f1000research.205102.r507440

**Published:** 2026-08-07

**Authors:** Edy Herianto Majlan

**Affiliations:** 1 Universiti Kebangsaan Malaysia, Bangi, Malaysia

This manuscript addresses a timely and policy-relevant question by explaining how renewable energy literacy may translate into multidimensional energy-saving behaviour in Indonesia. Its strengths are the integration of Value–Belief–Norm theory and the Theory of Planned Behaviour, the distinction among curtailment, efficiency, and pro-social actions, and the focus on an informed professional energy community. The model is motivated, the ethical procedures are transparently reported, and the findings offer useful implications for designing literacy programmes alongside economic instruments. With rigorous correction of the measurement, mediation, sampling, and hypothesis-testing issues identified below, the article could make a valuable contribution to behavioural energy research.

1. Ensure consistent use of either UK or US spelling: “behaviour” versus “behavior”.
2. Correct the Table 4 note from **β²** to **f²**.
3. Recheck the classification of f2=0.491f^2=0.491f2=0.491, which would normally be considered large rather than medium.
4. Clarify whether R² values in Table 4 belong to endogenous constructs rather than individual paths.
5. Provide exact p-values where practical, except values below the software reporting limit.
6. Add confidence intervals instead of relying only on t-values and p-values.
7. Define “pro-social energy action” more precisely.
8. Separate household behaviour from workplace or professional behaviour.
9. Reconsider the statement that removing the neutral midpoint reduces acquiescence bias. It may reduce midpoint selection but may also force respondents into positions they do not genuinely hold.
10. Review whether all references directly support the claims for which they are cited.
11. Correct statements describing the sample as representative of Indonesia’s “urban energy sector” unless urban location and occupational-sector evidence were collected.

Is the work clearly and accurately presented and does it cite the current literature?

Yes

If applicable, is the statistical analysis and its interpretation appropriate?

Yes

Are all the source data underlying the results available to ensure full reproducibility?

Yes

Is the study design appropriate and is the work technically sound?

Yes

Are the conclusions drawn adequately supported by the results?

Yes

Are sufficient details of methods and analysis provided to allow replication by others?

Yes

Reviewer Expertise:

Hydrogen Energy, Energy Management

I confirm that I have read this submission and believe that I have an appropriate level of expertise to confirm that it is of an acceptable scientific standard.
